# Supramolecular Diversity, Theoretical Investigation and Antibacterial Activity of Cu, Co and Cd Complexes Based on the Tridentate N,N,O-Schiff Base Ligand Formed In Situ

**DOI:** 10.3390/molecules27238233

**Published:** 2022-11-25

**Authors:** Elena A. Buvaylo, Oksana V. Nesterova, Evgeny A. Goreshnik, Hanna V. Vyshniakova, Svitlana R. Petrusenko, Dmytro S. Nesterov

**Affiliations:** 1 Department of Chemistry, Taras Shevchenko National University of Kyiv, Volodymyrska 64/13, 01601 Kyiv, Ukraine; 2 Centro de Química Estrutural, Institute of Molecular Sciences, Instituto Superior Técnico, Universidade de Lisboa, Av. Rovisco Pais, 1049-001 Lisbon, Portugal; 3 Department of Inorganic Chemistry and Technology, Jožef Stefan Institute, Jamova 39, 1000 Ljubljana, Slovenia; 4 L.V. Gromashevsky Institute of Epidemiology and Infectious Diseases NAMS of Ukraine, M. Amosova 5, 03038 Kyiv, Ukraine

**Keywords:** coordination compounds of Schiff base ligands, copper complexes, cobalt complexes, cadmium complexes, DFT and DLPNO-CCSD(T) calculations, QTAIM analysis of electron density, antibacterial activity

## Abstract

The four new complexes, [Cu(HL^1^)(L^2^)Cl] (**1**), [Cu(HL^1^)(L^1^)]∙Cl∙2H_2_O (**2**), [Co(L^1^)_2_]∙Cl (**3**) and [Cd(HL^1^)I_2_]∙dmso (**4**), have been prepared by one-pot reactions of the respective chloride or iodide metal salt with a non-aqueous solution of the polydentate Schiff base, HL^1^, resulted from in situ condensation of benzhydrazide and 2-pyridinecarboxaldehyde, while a ligand HL^2^, in case of **1**, has been formed due to the oxidation of 2-pyridinecarboxaldehyde under reaction conditions. The crystallographic analysis revealed that the molecular building units in **1**–**4** are linked together into complex structures by hydrogen bonding, resulting in 1D, 2D and 3D supramolecular architectures for **1**, **2** and **4**, respectively, and the supramolecular trimer for **3**. The electronic structures of **1**–**4** were investigated by the DFT theoretical calculations. The non-covalent interactions in the crystal structures of **1**–**4** were studied by means of the Hirshfeld surface analysis and the QTAIM theory with a special focus on the C–H⋯Cl bonding. From the DFT/DLPNO-CCSD(T) calculations, using a series of charged model {R_3_C–H}^0^⋯Cl^−^ assemblies, we propose linear regressions for assessment of the interaction enthalpy (Δ*H*, kcal mol^−1^) and the binding energy (BE, kcal mol^−1^) between {R_3_C–H}^0^ and Cl^−^ sites starting from the electron density at the bond critical point (*ρ*(**r**_BCP_), a.u.): Δ*H* = −678 × *ρ*(**r**) + 3 and BE = −726 × *ρ*(**r**) + 4. It was also has been found that compounds **1**, **3** and **4** during in vitro screening showed an antibacterial activity toward the nine bacteria species, comprising both Gram-positive and Gram-negative, with MIC values ranging from 156.2 to 625 mg/L. The best results have been obtained against *Acinetobacter baumannii* M*β*L.

## 1. Introduction

The Schiff bases are recognized as an important class of organic ligands for the design and construction of the polymeric and polynuclear coordination compounds since they can be easily formed as novel ligand systems using the simple in situ condensation of a large and variable library of readily available aldehydes and amines [1,2]. Such complexes present many applications in catalysis, magnetochemistry, oxygen storage devices and show antitumoral and antiviral and antibacterial activity [1,3,4,5]. The creation of effective antimicrobial agents is currently one of the most important challenges for medicinal chemistry. Due to the non-terminating development of the resistance of microorganisms (bacteria, fungi, viruses and parasites) to antimicrobial drugs, the search for new antimicrobial drugs still remains an essential and highly demanded task [6].

Nowadays, special attention is paid to the weak non-covalent interactions that often have predetermining role in the formation of supramolecular structures [7] as well as conformational changes in materials and bioactive molecules [8]. The molecular Schiff base complexes represent a good example for studying such types of intra- and intermolecular weak bonding due to the great diversity of Schiff base ligands [2]. The DFT theoretical calculation of the electron density and the extraction of its descriptors by means of quantum theory of atoms in molecules (QTAIM) provides a basis for the analysis of a weak interaction [9]. The search for the reliable correlation between the electron density at the bond critical points *ρ*(**r**_BCP_) and the bond energy is an open question. Emamian and Lu described simple linear regressions that allow an easy estimation of the binding energy from *ρ*(**r**_BCP_) for charged and neutral {X–H⋯Y} assemblies [10]. However, it is known that weak interactions (e.g., halogen bonding) are better described by regressions fitted to a specific data set rather than a broad one [11]. The C–H⋯Cl bonding is a case of particular interest because the respective contacts between chlorine-containing drugs and biological targets have a strong influence on the drug affinity and, therefore, the bioactivity [12,13,14]. Moreover, the interaction of a halide anion with C–H groups is the basis of many anion-selective receptors [12,15,16].

Earlier, we described the Schiff-based [Fe(HL^3^)Cl_2_(dmf)]Cl∙dmf (HL^3^ is formed from salicylic aldehyde and aminoethylpiperazine), [Cu(HL^4^)(NO_3_)(dmf)](NO_3_)∙H_2_O, [Cu(HL^4^)Cl_2_]∙0.5dmso (HL^4^ is formed from salicylaldehyde and 1-(2-aminoethyl)piperazine) and [CuCl_2_L^5^]·dmf (HL^5^ is formed from 2-pyridinecarbaldehyde and aminoguanidine) complexes which revealed the prominent catalytic activity in the cyclohexane oxidation with H_2_O_2_ in the presence of an acid promoter [17,18,19]. Moreover, it was found that homo-[CoL^6^_3_]·dmf and heterometallic [CoCdL^6^_3_Cl_2_]·0.5H_2_O and [CoZnL^6^_3_Cl_2_]·CH_3_OH (HL^6^ is formed from *o*-vanillin and methylamine) complexes demonstrate a profound catalytic activity in the stereoselective oxidation of *cis*-1,2-dimethylcyclohexane with *m*-chloroperbenzoic acid under mild conditions in the presence of promoters of different acidity [20,21]. Further, the heterometallic polynuclear [Co_4_Fe_2_O(L^7^)_8_]∙4dmf∙H_2_O complex (H_2_L^7^ is formed from salicylaldehyde and ethanolamine) shows exceptionally high activity in both catalytic systems mentioned above [22,23]. Apart from the catalytic studies, we previously described magneto-structural ones for the number of Schiff-based transition metal compounds, including [Fe_4_(µ_4_-O)_4_Mn_4_(L^8^)_8_(dmf)_4_]∙2dmf (H_2_L^8^ is formed from salicylaldehyde and hydroxylamine hydrochloride), [Cu_2_Fe_2_(HL^9^)_2_(H_2_L^9^)_2_]·10dmso, [Cu_2_Fe_2_(HL^10^)_2_(H_2_L^10^)_2_]·2dmf (H_2_L^9^ and H_2_L^10^ are formed from salicylaldehyde or 5-*bromo*-salicylaldehyde and tris(hydroxymethyl)aminomethane), [Cu_3_Mn(L^7^)_4_(CH_3_OH)_3_]I_3_, [Cu_3_Mn(L^7^)_4_(CH_3_OH)_3_(H_2_O)]NCS∙H_2_O, [Cu_3_Mn(L^7^)_4_(CH_3_OH)(H_2_O)_2.55_]Br∙0.45H_2_O, [Cu_3_Mn(L^7^)_4_(H_2_O)_3.4_]BF_4_∙0.6H_2_O, [Co_2_Fe_2_(L^11^)_6_]∙4dmf (H_2_L^11^ is formed from 5*-bromo-s*alicylaldehyde and 2-aminobenzyl alcohol) and [Co_4_Fe_4_(HL^12^)_8_(dmso)_2_]∙18dmso (H_4_L^12^ is formed from 5*-nitro-s*alicylaldehyde and tris(hydroxymethyl)aminomethane) complexes, revealed an antiferromagnetic coupling among the magnetic centers [24,25,26,27].

Continuing our research aiming at the synthesis and investigation of novel mono- and polynuclear transition metal complexes based on polydentate O,N-donor ligands [3,21,28,29,30,31,32,33], we have now synthesized four new complexes [Cu^II^(HL^1^)(L^2^)Cl] (**1**), [Cu^II^(HL^1^)(L^1^)]∙Cl∙2H_2_O (**2**), [Co^III^(L^1^)_2_]∙Cl (**3**) and [Cd^II^(HL^1^)I_2_]∙dmso (**4**) where HL^1^ is a product of in situ condensations of benzhydrazide and 2-pyridinecarboxaldehyde, HL^2^ is the 2-pyridinecarboxylic acid, studied their structural features, and tested the antibacterial activity toward the number of Gram-positive and Gram-negative bacteria species. Furthermore, we have investigated the electronic structures of the complexes **1**–**4** and have proposed new coefficients of linear regressions that allow the estimation of interaction enthalpies and binding energies for {C–H}^0^⋯Cl^−^ hydrogen bonds.

## 2. Results

### 2.1. Synthesis

**Scheme 1:**
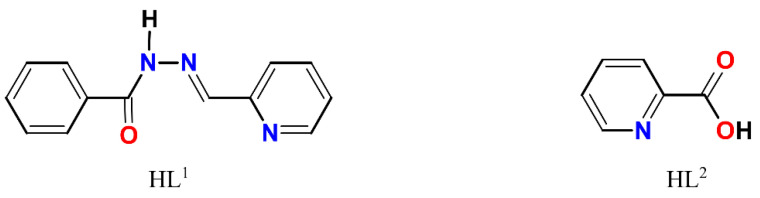
Structural formulae of HL^1^ and HL^2^.

The complexes **1**–**4** (Scheme S1) were obtained using the two steps synthetic approach within the same reaction vessel: (1) in situ formations of a Schiff base ligand by condensation of benzhydrazide and 2-pyridinecarboxaldehyde and (2) reaction of the prepared Schiff base ligand (HL^1^, Scheme 1) with a metal precursor. The choosing strategy receives a broad application in the synthesis of Schiff base-containing coordination compounds and allows to use of the Schiff base ligand immediately after its formation. The interaction of metal chlorides (**1**–**3**) or iodide (**4**) with a non-aqueous (CH_3_OH for **1**–**3** and dmso for **4**) solutions of HL^1^ using a molar ratio of MX_2_:HL^1^ = 1:2, resulted in green (**1**–**2**), brown (**3**) or yellow-orange (**4**) solutions obtained at the end of the reactions. All reactions were initiated and brought to completion by stirring and heating in the open air. Microcrystals **1** and **3** were formed in two days from the resulting solution, while microcrystals **2** were obtained from the filtrate of **1** in ca. one month. Microcrystals of **4** were obtained in two weeks after the addition of *^i^*PrOH into the resulting solution. Unexpectedly, in addition to the presence of desired HL^1^ Schiff base ligand in **1**, the single crystal X-ray analysis disclosed a presence of HL^2^, 2-pyridinecarboxylic acid (Scheme 1) as well. The formation of HL^2^ can be understood by assuming aerobic oxidation of 2-pyridinecarboxaldehyde under reaction conditions since this process is known for aldehyde chemistry [34]. The IR spectra of **1**–**4** and the ligand HL^1^ are depicted in Figures S1–S5.

### 2.2. Crystal Structures

**Figure 1:**
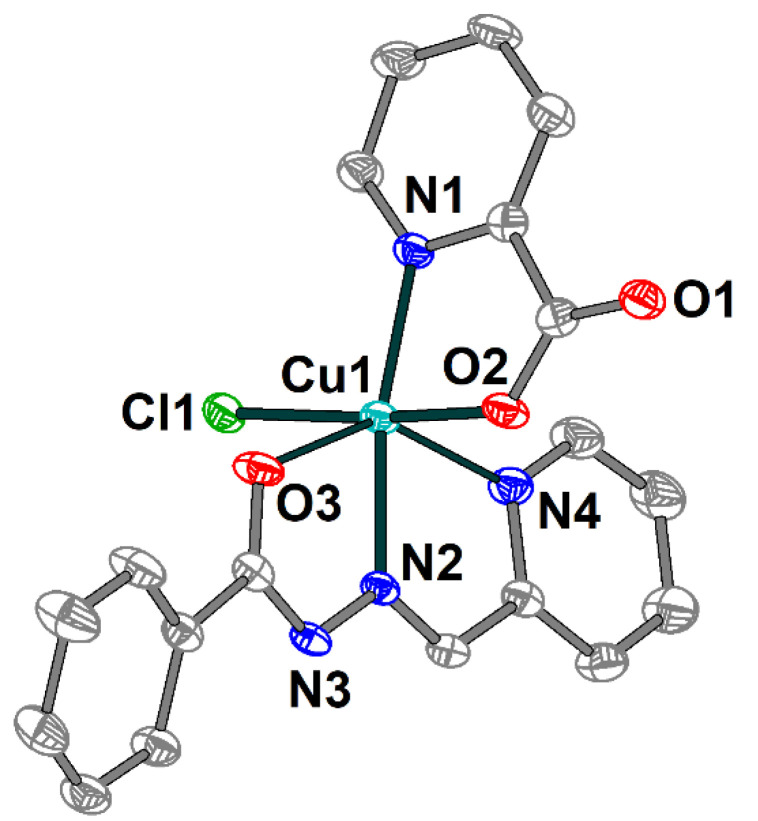
Molecular structure of **1** with the atom numbering scheme. H atoms are omitted for clarity. The atoms are shown with 50% probability displacement ellipsoids. Color scheme: Cu, cyan; O, red; N, blue; C, grey; Cl, green.

**Figure 2:**
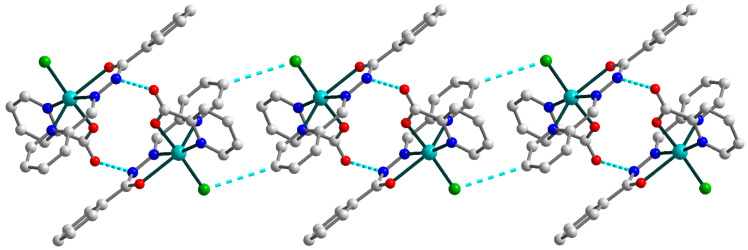
Representation of the supramolecular chain in **1**. H atoms are omitted for clarity. Color scheme: Cu, cyan; O, red; N, blue; C, grey; Cl, green.

The crystal structure of **1** consists of discrete mononuclear molecules [Cu(HL^1^)(L^2^)Cl] (Figure 1), which are jointed into supramolecular chains by means of hydrogen bonds (Figure 2). Complex **1** features two different ligands: the Schiff base ligand (HL^1^) showing tridentate N,N,O-donor coordination mode and the 2-pyridinecarboxylate (L^2^) coordinated in the N,O-donor fashion. The coordination polyhedron around the Cu(II) atom adopts O_2_N_3_Cl distorted elongated octahedral geometry formed by the O and N donor atoms of both organic ligands and coordinated chloride anion as well. The basal Cu–X (X = O, N, Cl) bond lengths in **1** vary from 1.9766(19) to 2.2591(7) Å, while the apical ones are 2.500(2) Å (Cu1–O3) and 2.378(2) Å (Cu1–N4) (Table S1). The X–Cu–N*_trans_* angles range from 144.84(7) to 173.00(6)°.

In the crystal lattice, the two closest neighboring molecules are strongly linked by hydrogen bonding between the oxygen atoms of the pyridine-carboxylates and the nitrogen atoms of Schiff base ligands (Figure 2) [N3–H3⋯O1^a^ (a = 1 − x, 1 − y, 1 − z), D–A = 2.884(3) Å, D–H⋯A = 150.1°]. Moreover, the hydrogen bonds formed with the participation of the Schiff base ligands and coordinated chloride anions [C8–H8⋯Cl1^b^ (b = 1 − x, 1 − y, −z), D–A = 3.734(3) Å, D–H⋯A = 153.9°] tie together abovementioned supramolecular dimers into one-dimensional chains. The nearest Cu⋯Cu distance within the supramolecular chain in **1** are 6.1899(10) and 8.1024(10) Å.

**Figure 3:**
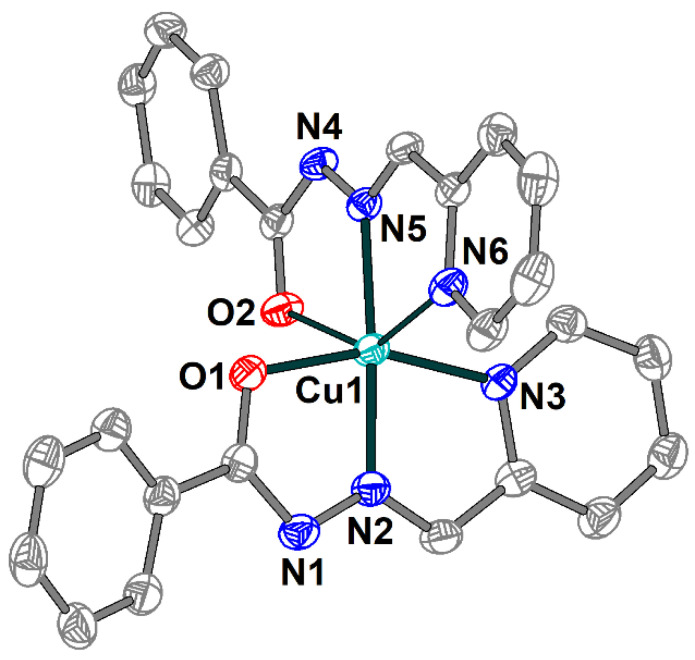
Structure of [Cu(HL^1^)(L^1^)]^+^ cation in **2** with the atom numbering scheme. H atoms, uncoordinated water molecules and chloride anion are omitted for clarity. The atoms are shown with 50% probability displacement ellipsoids. Color scheme: Cu, cyan; O, red; N, blue; C, grey.

The single crystal X-ray analysis shows that **2** includes a [Cu(HL^1^)(L^1^)]^+^ cation (Figure 3), one Cl^−^ anion and two uncoordinated water molecules joined into supramolecular two-dimensional layers assisted by hydrogen bonds. The Schiff base ligands in **2** are present in both deprotonated and non-deprotonated forms and reveal the tridentate-chelating (N,N,O) coordination mode. Similarly to **1**, the Cu(II) atom has a distorted elongated octahedral environment but with an O_2_N_4_ donor set formed by the Schiff bases. The Cu–X (X = O, N) bond lengths in the equatorial plain are in the range 1.931(2)–2.069(2) Å, and the apical Cu1–O2 and Cu1–N6 distances are 2.582(2) and 2.254(3) Å, respectively (Table S2). The O(N)–Cu–N*_trans_* bond angles vary from 144.32(8) to 175.14(10)°.

**Figure 4:**
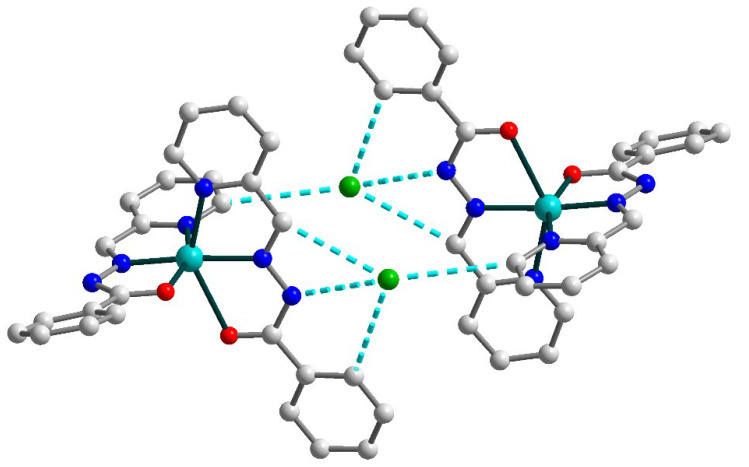
Supramolecular dimer in **2**. H atoms are omitted for clarity. Color scheme: Cu, cyan; O, red; N, blue; C, grey; Cl, green.

Due to the strong and multiple hydrogen bonds between uncoordinated chloride anions and donor atoms of Schiff base ligands [N4–H4⋯Cl1^b^ (b = 1 − x, 1 − y, −z), D–A = 3.245(3) Å, D–H⋯A = 160(4)°; C13–H13⋯Cl1, D–A = 3.675(3) Å, D–H⋯A = 165.5°; C20–H20⋯Cl1^b^, D–A = 3.645(3) Å, D–H⋯A = 173.5°; C21–H21⋯Cl1^b^, D–A = 3.457(3) Å, D–H⋯A = 140.0°], the formation of the supramolecular dimers in **2** can be identified (Figure 4). The non-bonded Cu⋯Cu separation within this dimer is 10.0391(9) Å.

**Figure 5:**
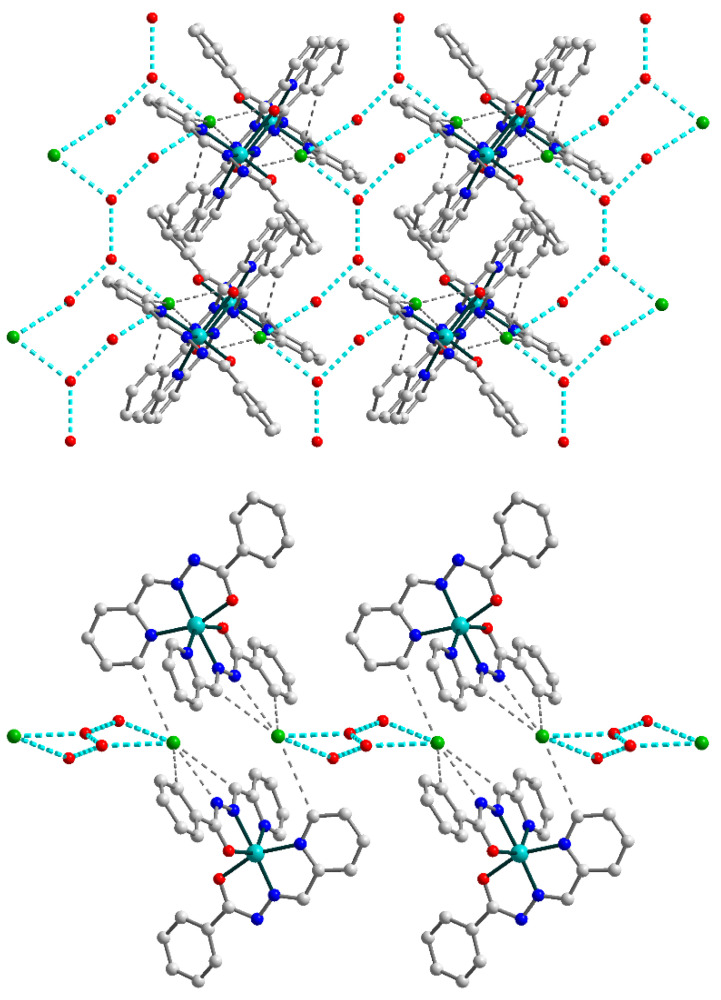
(**Top**) supramolecular 2D layer in **2**, showing supramolecular chains built by connected {O_H2O_⋯O_H2O_⋯Cl}_2_ synthons (highlighted by cyan dash lines). (**Bottom**) supramolecular layer in **2**, viewed down the crystallographic *a* axis. The hydrogen atoms are omitted for clarity. Color scheme: Cu, cyan; O, red; N, blue; C, grey; Cl, green.

Furthermore, the uncoordinated water molecules join the supramolecular dimers in **2,** forming two-dimensional layers by means of strong O–H⋯O and O–H⋯Cl hydrogen bonding between dimers and chloride anions (Figure 5) [O4A–H4AA⋯O3^b^ (b = 1 − x, 1 − y, −z), D–A = 2.721(9) Å, D–H⋯A = 150.3°; O4A–H4AB⋯Cl1, D–A = 3.288(8) Å, D–H⋯A = 151.5°; O3–H3A⋯Cl1, D–A = 3.315(3) Å, D–H⋯A = 166(6)°; O3–H3C⋯O3^c^ (c = –x, –y, –z), D–A = 2.795(7) Å, D–H⋯A = 166.38(11)°].

The chains formed by uncoordinated H_2_O molecules and Cl anions within the overall 2D structure of **2** can be considered as an independent supramolecular motif. They are built by {O_H2O_⋯O_H2O_⋯Cl}_2_ synthons which are connected through O–H⋯O bridges (Figure 5, top). Further increase of the dimensionality of the supramolecular 2D polymer is not observed due to steric factors: the bulky Schiff base ligands on the outer positions of the layer (Figure 5, bottom) prevent the formation of hydrogen bonds between neighboring sheets.

**Figure 6:**
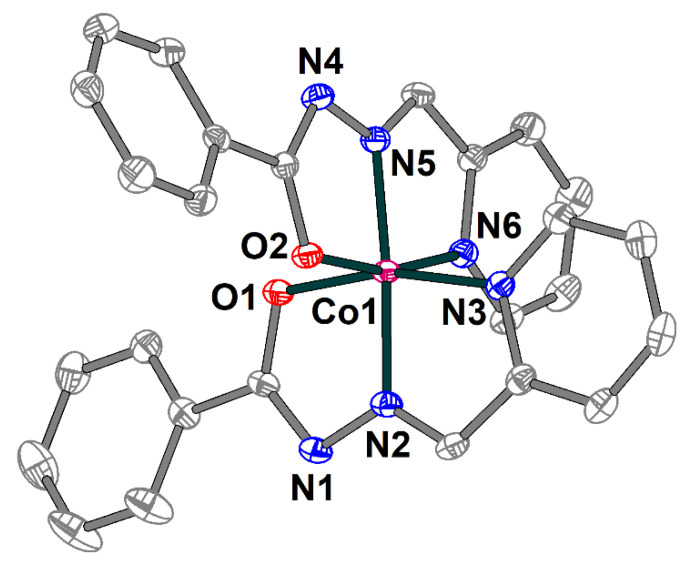
Structure of [Co(L^1^)_2_]^+^ cation in **3** with the atom numbering scheme. H atoms and chloride anion are omitted for clarity. The atoms are shown with 50% probability displacement ellipsoids. Color scheme: Co, dark pink; O, red; N, blue; C, grey.

**Figure 7:**
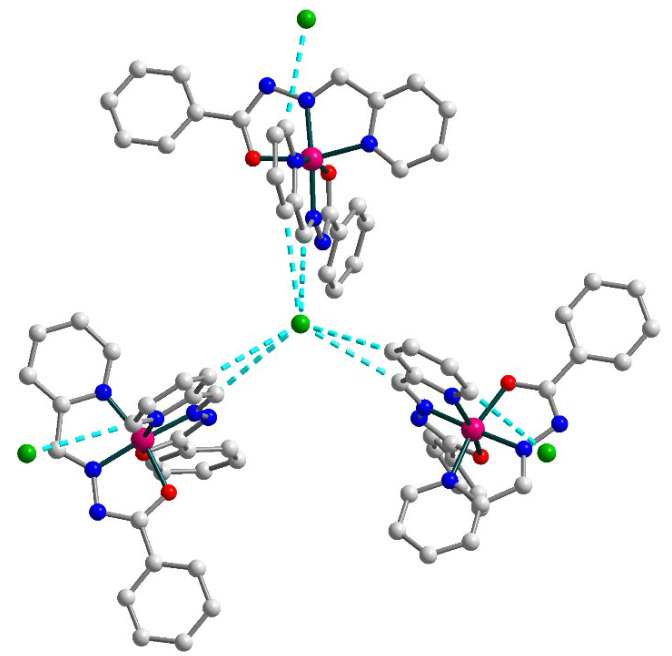
Supramolecular trimer in **3**. H atoms are omitted for clarity. Color scheme: Co, dark pink; O, red; N, blue; C, grey; Cl, green.

Complex **3** is built of [Co(L^1^)_2_]^+^ cations (Figure 6) and chloride anions, which are joined together by strong hydrogen bonding forming supramolecular trimers (Figure 7). The cobalt(III) atom in **3** has an octahedral coordination environment with an O_2_N_4_ donor set formed by two deprotonated Schiff base ligands. The Co–O and Co–N bond lengths are in the ranges 1.8927(13)–1.9099(14) and 1.8564(15)–1.9253(16) Å, respectively. The *trans* angles at the metal atom vary from 165.19(6) to 175.36(7), while the *cis* angles are equal to 82.35(6)–100.84(7)° (Table S3).

In the solid state, the [Co(L^1^)_2_]^+^ cations with uncoordinated Cl anions form supramolecular trimers by means of C–H⋯Cl bonding (Figure 7) [C21–H21⋯Cl1, D–A = 3.622(2) Å, D–H⋯A = 141.6°; C23–H23⋯Cl1, D–A = 3.571(2) Å, D–H⋯A = 150.8°]. Additionally, one more chlorine anion is connected by C–H⋯Cl contacts to each cobalt(III) moiety of this trimer [C26–H26⋯Cl2A, D–A = 3.480(4) Å, D–H⋯A = 154.6°]. The observed Co⋯Co intermolecular separation within the supramolecular trimer is 10.397 Å.

**Figure 8:**
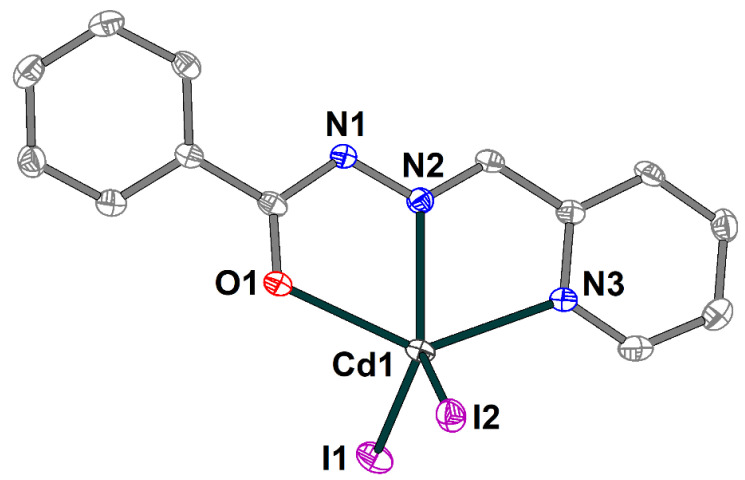
Molecular structure of **4** with the atom numbering scheme. H atoms and solvate dmso molecules are omitted for clarity. The atoms are shown with 50% probability displacement ellipsoids. Color scheme: Cd, medium grey; O, red; N, blue; C, grey; I, violet.

**Figure 9:**
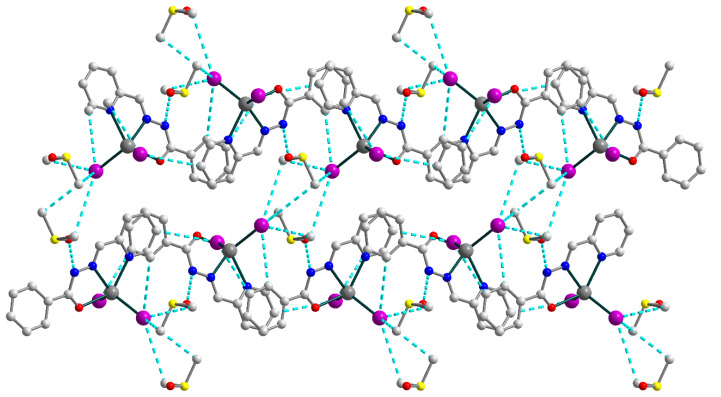
Supramolecular 3D structure in **4**, viewed down the crystallographic *a* axis. The hydrogen atoms are omitted for clarity. Color scheme: Cd, medium grey; O, red; N, blue; C, grey; I, violet; S, yellow.

The crystal structure of the **4** is formed of discrete neutral [Cd(HL^1^)I_2_] molecules (Figure 8) and dmso molecules of crystallization, joined by strong hydrogen bonding with the formation of an extended supramolecular 3D structure (Figure 9). The Schiff base ligand in **4** is in a neutral form and shows the tridentate-chelating (N,N,O) coordination fashion, similar to **1**–**3**. The Cd(II) atom is five-coordinated and has an ON_2_I_2_ donor set. The Cd–X (X = O, N, I) bond lengths fall in the range of 2.314(3)–2.7209(4) Å (Table S4), while the angles at the Cd atoms vary from 66.86(9) to 135.73(9)°.

The strong N–H⋯O and C–H⋯I hydrogen bonds [N1–H1⋯O2, D–A = 2.717(4) Å, D–H⋯A = 147.0°; C3–H3⋯I1^d^ (d = x, 0.5 − y, −0.5 + z), D–A = 4.142(4) Å, D–H⋯A = 152.3°; C4–H4⋯I2^e^ (e = −1 + x, 0.5 − y, −0.5 + z), D–A = 4.004(2) Å, D–H⋯A = 159.4°; C12–H12⋯I2^d^, D–A = 3.964(4) Å, D–H⋯A = 148.6°; C14–H14B⋯I1^e^, D–A = 4.283(4) Å, D–H⋯A = 154.8°; C14–H14C⋯I1^f^ (f = 1 − x, −0.5 + y, 1.5 − z), D–A = 4.180(4) Å, D–H⋯A = 147.7°; C15–H15A⋯I1^f^, D–A = 4.112(4) Å, D–H⋯A = 160.8°; C15–H15B⋯I1^e^, D–A = 4.139(4) Å, D–H⋯A = 158.2°], involving nitrogen atoms from imine moieties of Schiff base ligands, oxygen atoms from uncoordinated dmso molecules, carbon atoms from Schiff bases and dmso molecules and coordinated I^−^ anions, are responsible for the formation of the extended supramolecular three-dimensional framework (Figure 9). The closest Cd⋯Cd intermolecular separation within the framework exceeds 7.48 Å.

### 2.3. Theoretical Calculations

**Figure 10:**
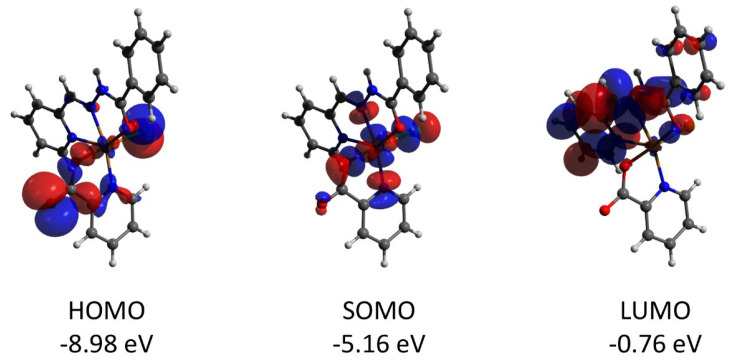
Plots and energies of the frontier quasi-restricted molecular orbitals (shown at 0.01 a.u. isosurface) of **1**, calculated at the ωB97X-D4/ma-def2-TZVP level and based on crystallographic atom coordinates. Colour scheme: Cu, orange; O, red; N, blue; C, grey; Cl, green.

The mononuclear complex **1** represents a “classical” Cu(II) coordination compound with the *S* = 1/2 ground state and unpaired electron localized at the copper center (spin population of 0.710, according to the Löwdin population analysis of the quasi-restricted molecular orbitals). The HOMO orbital has large contributions from *p*_z_ (30.1%) and *p*_x_ (19.0%) orbitals of the oxygen atom and *p*_z_ (17.4%) of the chlorine one (Figure 10). The LUMO orbital is strongly delocalized over the atoms of the ligands.

**Figure 11:**
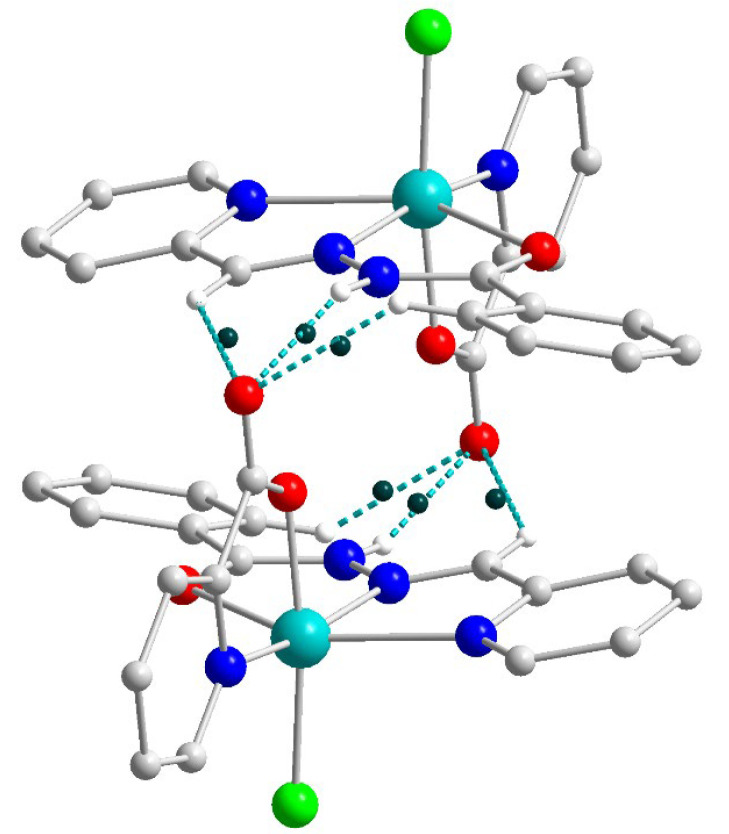
Structure of the supramolecular dimer in **2** showing the bond critical points (BPCs, dark-green dots) for the set of six strong hydrogen bonds. Color scheme: Cu, cyan; O, red; N, blue; C, grey; Cl, green; H, white.

In the supramolecular structure of **1** (Figure 2), one can select strongly bridged dimers, strengthened by the N–H⋯O and C–H⋯O hydrogen bonds as well as the π–π stacking. Analysis of the electron density ρ(**r**) and the bond critical points (BCPs) reveals two symmetrically equivalent sets of critical points (Figure 11), both formed by the oxygen atom O1 and H^a^ atoms from N3^a^, C12^a^ and C19^a^ ones (where the symmetry operation ^a^ is 1 − x, 1 − y, 1 − z). The sum electron density ρ(**r**) in these six BCPs is 7.1 × 10^−2^ a.u. According to the model developed by Emamian and Lu [10], this value corresponds to the binding energy of −15.1 kcal mol^−1^, which can be considered a strong H-bonded coupling.

**Figure 12:**
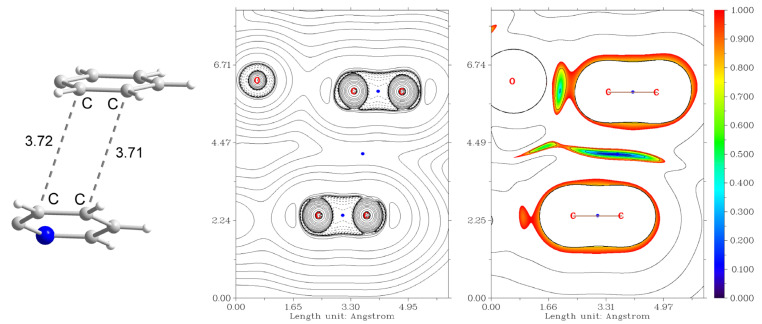
Non-covalent interaction between the phenyl and pyridine rings of the Schiff base ligands in the supramolecular dimer of **1**. (**Left**) the fragment of the structure showing the C⋯C distances. (**Centre**): plot of the Laplacian of electron density ∇^2^*ρ*(**r**), where BCPs are shown as blue dots. (**Right**) plot of the reduced density gradient (RDG) [36] isosurfaces showing attraction (blue), van der Waals (green) and repulsion (red) interactions. Color scheme: N, blue; C, grey; H, white.

The additional contribution to the binding energy of −1 kcal mol^−1^ comes from the interaction of phenyl and pyridine rings of the Schiff base ligands (Figure 12). The aromatic rings interact in the distorted parallel offset mode [35] with the angle of 25.4° and ρ(**r**_BCP_) = 3.9 × 10^−3^ a.u. for the closest contacts. Optimization of the geometry of the dimer of **1** reveals that the parallel offset mode persists (Figure S6), while the angle between planes formed by the aromatic rings becomes much smaller (10.0°). This suggests that rotation of the aromatic rings in the solid state is induced by the intermolecular interactions, not accounted for in the molecular geometry optimization.

The broken symmetry calculations revealed the very small singlet-triplet splitting of −0.05 and −0.12 cm^−1^ (using B3LYP and ωB97X-D4 functionals, respectively, *H* = *JS*_1_*S*_2_ notation and *J* = 2(*E*_HS_ − *E*_BS_)/(*S*_A_ + *S*_B_)^2^ formalism [37,38,39]). The calculated overlap between the magnetic orbitals (according to the analysis of unrestricted corresponding orbitals), containing unpaired electrons located on the copper atoms, constitutes 0.004 and 0.001 for B3LYP and ωB97X-D4 functionals, respectively. These values are ca. one order smaller than those expected for spin-coupled orbitals [40]. Thus, one can conclude that Cu(II) centers in **1** are almost magnetically isolated, but the existence of a small ferromagnetic coupling cannot be excluded.

**Figure 13:**
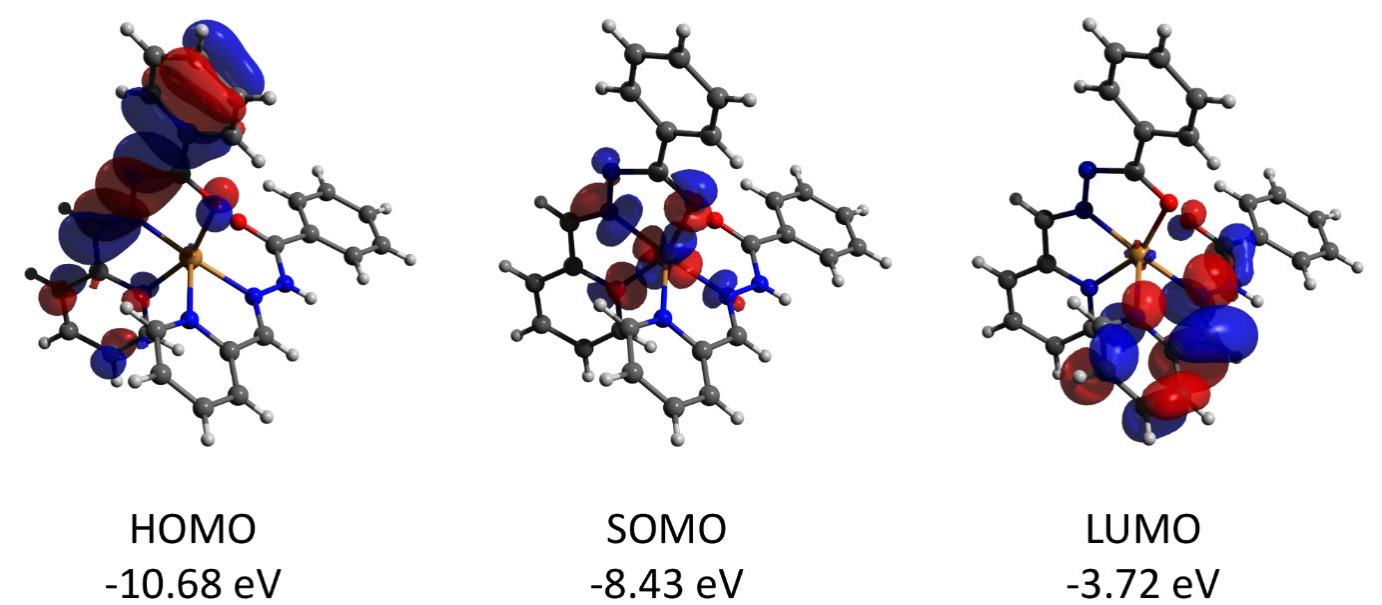
Plots and energies of the frontier quasi-restricted molecular orbitals (shown at 0.01 a.u. isosurface) of the cation of **2**, calculated at the ωB97X-D4/ma-def2-TZVP level and based on crystallographic atom coordinates. Color scheme: Cu, orange; O, red; N, blue; C, grey.

**Figure 14:**
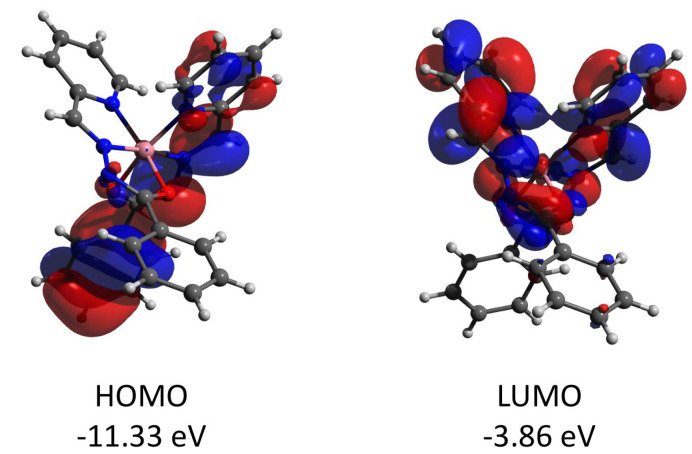
Plots and energies of the frontier molecular orbitals (shown at 0.03 a.u. isosurface) of the cation of **3**, calculated at the ωB97X-D4/ma-def2-TZVP level and based on crystallographic atom coordinates. Color scheme: Co, pink; O, red; N, blue; C, grey.

**Figure 15:**
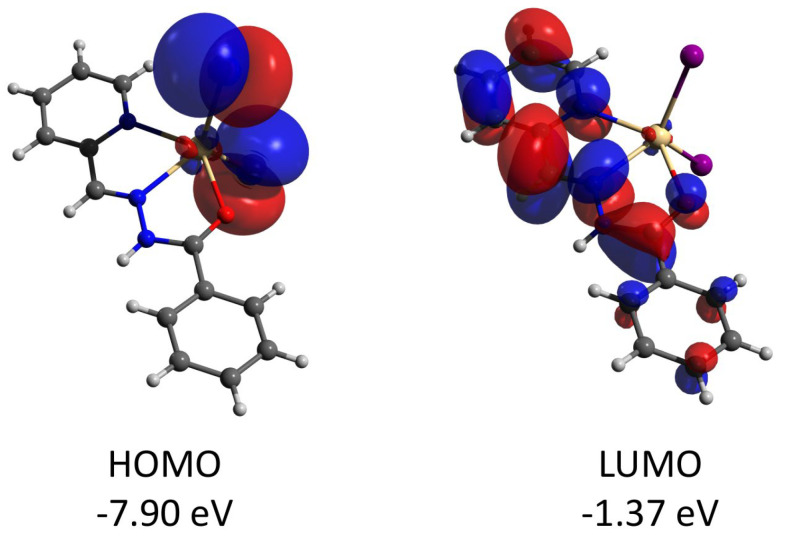
Plots and energies of the frontier molecular orbitals (shown at 0.03 a.u. isosurface) of **4**, calculated at the ωB97X-D4/ma-def2-TZVP level and based on crystallographic atom coordinates. Color scheme: Cd, pale yellow; O, red; N, blue; C, grey; I, violet.

Complex **2** features the HOMO and LUMO quasi-restricted molecular orbitals strongly delocalized over the ligands’ atoms (Figure 13). The spin population (*S* = 1/2 ground state) of the copper center is similar (0.727) to that found for complex **1**. The HOMO orbital of the diamagnetic complex **3** is totally located on one of the ligands, while the LUMO orbital is delocalized over two ligand moieties (Figure 14). Both HOMO and LUMO molecular orbitals in the cation of **3** have negligible (less than 1%) contributions to the atomic orbitals of the cobalt atom. The same as for **3**, the HOMO molecular orbital of the diamagnetic complex **4** is located at the halogen atoms and does not involve the cadmium center (Figure 15). Both iodine atoms have a high contribution, namely 39.1% of *p*_x_ orbital of I(1) and 22.8% *p*_z_ and 22.1% of *p*_z_ and *p*_y_, respectively, of I(2).

**Figure 16:**
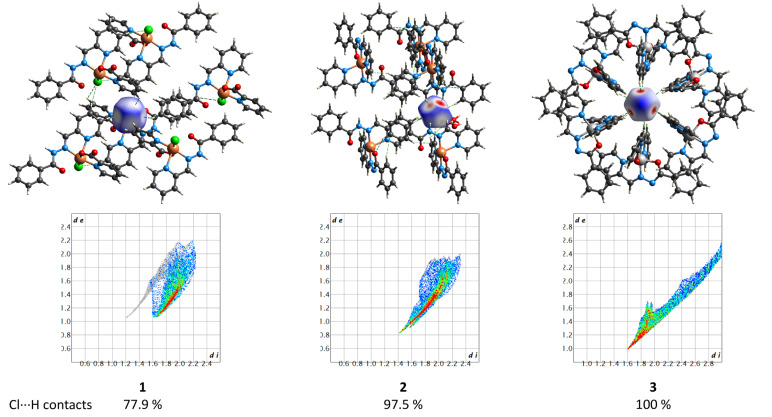
(**Top**) the Hirshfeld surfaces of chloride anions in **1**–**3**. The colored map corresponds to a normalized contact distance (*d*_norm_). (**Bottom**) the respective fingerprint plots (*d*_e_ vs. *d*_i_, Å). Color scheme: Cu, orange; Co, light grey; O, red; N, blue; C, grey; Cl, green.

**Figure 17:**
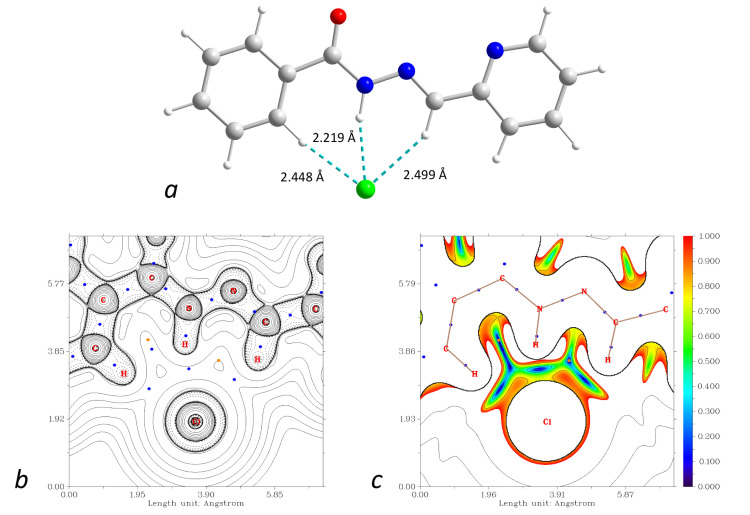
(**a**): DFT-optimized structure of the {HL^1^·Cl}^−^ assembly. (**b**): plot of the Laplacian of electron density ∇^2^*ρ*(**r**) highlighting the Cl⋯H interactions (BCPs are shown as blue dots). (**c**): plot of the reduced density gradient (RDG) at the same plane. Color scheme: Cu, cyan; O, red; N, blue; C, grey; Cl, green; H, green.

The supramolecular structures of the complexes **1**–**3** reveal numerous C–H⋯Cl contacts of various strengths, where coordinated (**1**) or free (**2** and **3**) chloride anions are surrounded by the hydrogen atoms from the ligands. The Hirshfeld surface analysis [41] and the fingerprint plots disclose that the Cl⋯H contacts constitute the largest contribution to the intramolecular Cl⋯X interactions, being 100% for **3** (Figure 16). The non-deprotonated form of the ligand (HL^1^) is an excellent receptor for the anions due to the presence of three C–H groups directed approximately to a single point. Geometry optimization of the {HL^1^·Cl}^−^ assembly using the coordinates from structure **2** as the starting point shows that the chlorine atom is in the ligand’s plane, forming three strong hydrogen bonds (Figure 17). The enthalpy of the interaction between HL and Cl^−^ was found to be −32.1 kcal mol^−1^ (ωB97X-D4/ma-def2-TZVP level).

It is known that the energies of weak interactions (including hydrogen bonds) can be approximately estimated through the evaluation of the electron density *ρ*(**r**) at the respective bond critical points (BCPs). This approach allows for the extraction of the energies of intramolecular interactions for which the individual energies of donor and acceptor sites cannot be calculated due to the inseparability of the whole molecule. While the BE vs. *ρ*(**r**) linear regressions (where BE is the binding energy) proposed by Emamian and Lu can be applied to a broad range of hydrogen bonds [10], re-examination of the BE vs. *ρ*(**r**) reference data for a certain X–H⋯Y pair may result in more precise coefficients of regression.

The choice of a calculation method for precise estimation of interaction energies in weakly bonded supramolecular assemblies is a topic of broad discussion, where the computational resources required to perform calculations at high levels are a crucial factor [42]. We selected several examples from the S66 dataset of the Benchmark Energy & Geometry Database (BEGDB) [43], where small molecules (such as water and methanol) form weak hydrogen bonds with other fragments. The original binding energies reported by Hobza et al. [43] were reconsidered several times [44,45]. Herein we used the BEs reported by Martin et al. [45] as a reference, where energies were calculated using a few sets of explicitly correlated methods with the basis sets near the limit along with counterpoise (CP) and other corrections. Since the accurate geometries and interaction energies in S66 were accurately determined [43,45], we tested several routine methods against the models **01**, **03**, **04**, **09** and **18** of the S66 dataset. The geometries re-optimized at the ωB97X-D4/ma-def2-TZVP level showed only a slight difference from those reported in S66 (Table S8). The lowest deviation was found for the model **01** (HOH⋯OH_2_ water dimer), while the highest one for the model **09** (interaction of methanol with methylamine). For these re-optimized geometries, we calculated the binding energies using the DLPNO-CCSD(T) scheme involving def2-TZVPPD and aug-cc-pV{D,T}Z, aug-ano-pV{D,T}Z and aug-ano-pV{T,Q}Z basis sets [46,47,48] with or without counterpoise correction [49] (Table S8). The extrapolated aug-ano-pV{T,Q}Z CP-corrected basis set gave excellent (<0.08 kcal mol^−1^) agreement with the reference data for **01** and **03** models (RMS = 0.09). However, this method is computationally demanding, and its use for heavier models was not justified under our conditions. Surprisingly, the second method of high accuracy was def2-TZVPPD without CP correction, which showed an RMS of 0.125 and the highest deviation of 0.21 kcal mol^−1^ (Table S8). A similar level of accuracy was observed for extrapolated aug-cc-pV{D,T}Z and aug-ano-pV{D,T}Z basis sets involving CP-correction (RMS = 0.161 and 0.182, respectively), while all extrapolated methods showed considerably higher RMS in the absence of counterpoise correction. Remarkably, the application of counterpoise correction along with the def2-TZVPPD basis set resulted in the highest RMS of 0.66 and BE deviation of almost 1 kcal mol^−1^. It is known that, in certain cases, the errors of smaller basis sets may compensate for basis set superposition error (BSSE) and other systematic errors. For example, Antony and Grimme recommended SCS-MP2 with the triple-ζ basis sets (TZVPP and cc-pVTZ) without CP correction as the methods give reasonable results for large systems involving weak interactions [50]. In some cases, the half-counterpoise correction [10,51] or extrapolation to the basis set limit was found to better describe the reference data [52,53]. Although the BSSE largely affects the small (double-ζ) basis sets [54,55], the DFT scheme used herein for geometry optimizations (ωB97X-D4/ma-def2-TZVP) may suffer from the BSSE when calculating binding energies [54], also because estimation of the absolute electronic energy is a known weak point of the DFT. However, the geometrical parameters of the S66 models calculated at the DFT level are close to those obtained at the *ab initio* level (Table S8), while the DLPNO-CCSD(T) correction of the final energy allows approaching the reference BE values.

We checked how the H⋯Cl separation in the {CH_4_⋯Cl}^−^ model assembly is influenced by the calculation level (Table S9). The ωB97X-D4/ma-def2-TZVP method affords *d*(H⋯Cl) = 2.689 Å. The application of the geometrical semi-empirical counterpoise correction (gCP) [56] along with the def2-TZVP basis set shortens the *d*(H⋯Cl) distance down to 2.591 Å (the pure def2-TZVP calculation gives *d*(H⋯Cl) = 2.575 Å), indicating that diffuse functions are essential for the present case of anion assembly. The increase of the basis set up to def2-QZVPPD, which is large enough to minimize BSSE until negligible level [57,58], results in H⋯Cl separation of 2.672 Å, which is slightly shorter than that for ma-def2-TZVP. A very similar distance of 2.673 Å was obtained for a very large aug-cc-pV6Z basis set (Table S9). While the ωB97M-V [59] and M06-2X [60] functionals showed similar tendencies, the SCS-MP2 method [61] gave much shorter *d*(H⋯Cl) = 2.603 Å for aug-cc-pV6Z, while for def2-QZVPPD the elongated distance of 2.713 Å was obtained. Finally, we optimized the {CH_4_⋯Cl}^−^ geometry at the DLPNO-CCSD(T)/def2-QZVPPD level, which gave *d*(H⋯Cl) = 2.651 Å (Table S9). Thus, the geometry exhibited by the ωB97X-D4/ma-def2-TZVP method fits into the spread of *d*(H⋯Cl) distances. Considering this, as well as the success in the description of the selected S66 models (Table S8), we assume the precision of this DFT method to be sufficient for the present study.

**Figure 18:**
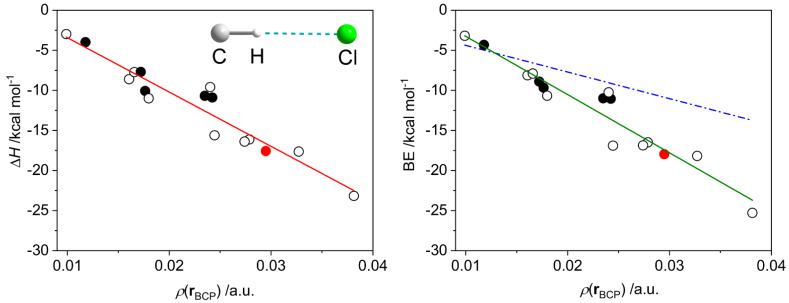
Dependences of the dissociation enthalpy (Δ*H*, **left**) and the binding energy (BE, **right**) on the electron density *ρ*(**r**) at the H⋯Cl bond critical point in the set of model compounds featuring one (empty and black filled circles) or two (red filled circle) {R_3_C–H}^0^⋯Cl^−^ hydrogen bonds (Figure S8), where Cl is an isolated atom. The black-filled circles correspond to the radical species (triplet state); all others are in the singlet state. Solid red and green lines correspond to the least-squares linear fits: Δ*H* = −678 × *ρ*(**r**) + 3 and BE = −726 × *ρ*(**r**) + 4. Dash dot blue line (**right**) was calculated using the parameters reported by Emamian and Lu [10].

We considered a series of model complexes {R_3_C–H}^0^⋯Cl^−^, including radical forms (Tables S5 and S6, Figures S8–S10), for which BE vs. *ρ*(**r**) and Δ*H* vs. *ρ*(**r**) dependences were studied. Both binding energy and enthalpy data can be fitted to linear equations with *R*^2^ of 0.92 and 0.91 and the RMS values of 1.60 and 1.55, respectively (Figure 18).

The slope of the fitted line obtained for the BE vs. *ρ*(**r**) data (−726) significantly differs from that proposed by Emamian et al. for charged complexes (−332.34) [10]. Moreover, the latter equation is not able to describe the data in the region of strong interactions (Figure 18, right). To ensure that this discrepancy is not a result of differences in calculation methodology, we reproduced the geometries, electron densities and binding energies of several H-bonded assemblies used in [10]. We found that under our conditions, the energies of the assemblies and the slope of the BE vs. *ρ*(**r**) linear fit are almost identical to those reported by Emamian et al. [10] (Figure S11). Therefore, one can conclude that the case of {R_3_C–H}^0^⋯Cl^−^ interactions is better described by the linear regression proposed herein. Further, we did not find a notable change in enthalpies when using larger basis sets (ma-def2-QZVPP and aug-cc-pVQZ) for DLPNO-CCSD(T) correction (Figure S12). The pure DFT calculations (without the DLPNO-CCSD(T) correction) give the binding energies only slightly different from those for CCSD-corrected DFT data, maintaining the overall statistical distribution (Figure S13).

From the proposed regressions, the interaction enthalpy of the {HL^1^·Cl}^−^ assembly (Figure 17) is expected to be –37.6 kcal mol^−1^, which is rather close to the value found from the vibrational energies at the DFT level (−32.1 kcal mol^−1^). As the {HL^1^·Cl}^−^ constitutes two C–H⋯Cl and one N–H⋯Cl bonds, we checked the applicability of the proposed model for {N–H}^0^⋯Cl^−^ contacts: the DFT and regression model predicted Δ*H* for {CH_3_NH_2_⋯Cl}^−^ equal to −9.8 and −9.7 kcal mol^−1^, respectively. For the protonated ligand {H_2_L^1^}^+^ interacting with the chloride anion, the regression predicts Δ*H* = −58.6 kcal mol^−1^, while the DFT calculations give twice higher value Δ*H* of −112.8 kcal mol^−1^. Such a strong interaction is illustrated by significant shortening of the N–H⋯Cl contact from 2.219 to 1.857 Å for {HL^1^·Cl}^−^ and {H_2_L^1^·Cl}^0^, respectively (Figure 17 and Figure S7), where the Löwdin charges at the respective H atoms in the free ligands are 0.219 and 0.241, respectively. Therefore, the linear regressions proposed herein should be used with care for the assessment of the stability of supramolecular assemblies with charges other than {R_3_C–H}^0^⋯Cl^−^.

### 2.4. Antibacterial Activity Studies

Complexes **1**, **3** and **4** were screened for antibacterial activity with a following evaluation of the minimal inhibitory concentration (MIC) (Table 1) toward the panel of reference microorganisms from the American Type Culture Collection (ATCC), including Gram-negative bacteria (*Escherichia coli* ATCC 25922, *Pseudomonas aeruginosa* ATCC 27853, *Klebsiella pneumoniae* ATCC 700603, *Acinetobacter baumannii* ATCC BAA 747, *Pseudomonas aeruginosa* HUI PAM*β*L) and Gram-positive bacteria (*Staphylococcus aureus* ATCC 25923, *Staphylococcus aureus* HUI MRSA, *Staphylococcus aureus* ATCC MR 43300, *Staphylococcus haemolyticus* HUI MRCNS).

The MIC values ranged between 156.25 and 312.5 mg/L for compounds **1** and **4** and varied in the range of 156.2–625 mg/L for compound **3**. Among the strains of Gram-negative bacteria, the best results were obtained in the case of *Acinetobacter baumannii* ATCC BAA 747, where all three studied compounds show the lowest observed MIC value of 156.25 mg/L. The other tests involving Gram-negative bacteria mostly show the moderate antibacterial activity of **1**, **3** and **4** with MIC of 312.5 mg/L, while the lowest activity was documented in the case of **3** toward *Pseudomonas aeruginosa* HUI PAM*β*L (625 mg/L). The most active compound in the experiments with a group of Gram-positive bacteria was complex **4**, showing the MIC value of 156.25 mg/L in the case of three out of four studied strains. Compounds **1** and **3** reveal moderate activity toward Gram-positive bacteria with an MIC value of 312.5 mg/L, except for tests including *Staphylococcus aureus* ATCC 25923 and compound **3**, which shows MIC equal to 156.25 mg/L. The obtained results indicate that all studied compounds show a broad spectrum of activity against the tested microorganisms and reveal relatively better activity against Gram-positive than Gram-negative bacteria, and the best activity was observed against *Acinetobacter baumannii* ATCC BAA 747.

Comparing the results obtained for **1** with those reported previously for Cu(II) complexes, one can observe that in the case of Gram-positive bacteria *Staphylococcus aureus* ATCC 25923 the complex **1** showed lower antibacterial activity (MIC of 312.5 mg/L) than the compound [Cu(ClNBz)(*o*-PDA)]Cl (where ClNBz is 4-chloro-3-nitrobenzoate and *o*-PDA is *o*-phenylenediamine) [62] revealing a MIC of 39 mg/L. However, in the case of Gram-negative bacteria *Pseudomonas aeruginosa* ATCC 27853, compound **1** performed much better, showing a MIC value of 312.5 mg/L in comparison with a MIC of 625 mg/L of [Cu(ClNBz)(*o*-PDA)]Cl [62]. The Cu(II) complexes CuL_1_A and CuL_2_A (where HL_1_A and HL_2_A are asymmetrical Schiff base ligands) [63] have the same MIC value of 128 mg/L against the tested Gram-positive (*Staphylococcus aureus* ATCC 25923) and Gram-negative (*Escherichia coli* ATCC 25922 and *Pseudomonas aeruginosa* ATCC 27853) bacteria, while **1** showed MIC = 312.5 mg/L in all similar tests. The CuCl_2_ is known to have the same as **1** antibacterial activity toward *Staphylococcus aureus* ATCC 25923 (MIC of 312 mg/L) and higher than **1** activity toward *Pseudomonas aeruginosa* ATCC 27853 (MIC of 156 mg/L) [62]. The MIC value of 272 mg/L was determined during the antibacterial study of the Co(II) complex [Co(H_2_BPClNOL)Cl_2_] (where H_2_BPClNOL is N-(2-hydroxybenzyl)-N-(2-pyridylmethyl)[(3-chloro)(2-hydroxy)]propylamine) [64] against the Gram-positive bacteria *Staphylococcus aureus* ATCC 25923. This value is higher than the respective MIC value of **3** (156.25 mg/L) and indicates a lower activity of [Co(H_2_BPClNOL)Cl_2_] in such a test. Similarly to **1**, **3** and **4**, the Ni(II) complex, [Ni_3_(C_10_H_11_NO_2_S)_3_]·C_3_H_7_NO [65], showed the highest activity with the lowest minimum inhibitory concentration value of 156.2 mg/L against Gram-negative bacteria *Acinetobacter baumannii* ATCC BAA 747.

## 3. Experimental

All chemicals were of reagent grade and used as received. All chemical experiments were carried out in air. Elemental analyses for CHN were provided by the Microanalytical Service of the Taras Shevchenko National University of Kyiv. Infrared spectra (4000–400 cm^−1^) were recorded in KBr pellets using a PerkinElmer 1600 FT-IR (PerkinElmer Inc., Waltham, MA, USA) instrument.

### 3.1. Syntheses of [Cu(HL^1^)(L^2^)Cl] (1) and [Cu(HL^1^)(L^1^)]∙Cl∙2H_2_O *(**2**)*

Benzhydrazide (0.27 g, 2 mmol) and 2-pyridinecarboxaldehyde (0.19 mL, 2 mmol) were dissolved in CH_3_OH (20 mL), forming a light-yellow solution which was magnetically stirred at 50–60 °C (30 min). Then, CuCl_2_·2H_2_O (0.17 g, 1 mmol) was added, and the resulting mixture was stirred for 1 h. The brown precipitate, which was immediately formed after cooling the obtained solution, was filtered, and the resulting solution was kept at r.t. Dark green crystals of **1**, suitable for the X-ray crystallographic study, were formed in two days. Yield: 0.1 g. Anal. calc. for C_19_H_15_ClCuN_4_O_3_ (M = 446.34): C, 51.13; H, 3.39; N, 12.65%. Found: C, 51.4; H, 3.4; N, 12.6%. Further, the obtained filtrate was kept at r.t., and dark green crystals of **2** suitable for X-ray crystallographic study were formed in one month. Yield: 0.06 g (the total yield of **1** and **2** is 33%). Anal. calc. for C_26_H_25_ClCuN_6_O_4_ (M = 584.51): C, 53.42; H, 4.31; N, 14.38%. Found: C, 53.4; H, 4.0; N, 14.7%.

### 3.2. Synthesis of [Co(L^1^)_2_]∙Cl *(**3**)*

Benzhydrazide (0.27 g, 2 mmol) and 2-pyridinecarboxaldehyde (0.19 mL, 2 mmol) were dissolved in CH_3_OH (20 mL), forming a light-yellow solution which was magnetically stirred at 50–60 °C (30 min). Then, CoCl_2_·6H_2_O (0.24 g, 1 mmol) was added, and the resulting mixture was stirred for 1 h. Dark red crystals of **3**, suitable for the X-ray crystallographic study, were formed in two days. Yield: 0.33 g, 60%. Anal. calc. for C_26_H_20_ClCoN_6_O_2_ (M = 542.86): C, 57.52; H, 3,71; N, 15.48%. Found: C, 57.6; H, 3.9; N, 15.2%.

### 3.3. Synthesis of [Cd(HL^1^)I_2_]∙dmso *(**4**)*

Benzhydrazide (0.27 g, 2 mmol) and 2-pyridinecarboxaldehyde (0.19 mL, 2 mmol) were dissolved in dmso (20 mL), forming a light-yellow solution which was magnetically stirred at 50–60 °C (30 min). Then, CdI_2_ (0.37 g, 1 mmol) was added, and the resulting mixture was stirred for 2 h. Yellow-orange crystals of **4**, suitable for X-ray crystallographic study, were formed two weeks after the addition of 4 mL of *^i^*PrOH. Yield: 0.14 g, 21%. Anal. calc. for C_15_H_17_CdI_2_N_3_O_2_S (M = 669.58): C, 26.91; H, 2.56; N, 6.28%. Found: C, 26.7; H, 2.3; N, 5.9%.

### 3.4. Crystallography

Single-crystal X-ray data for all compounds were collected on a Gemini A diffractometer (Agilent Technologies Inc., Santa Clara, CA, USA) equipped with an Atlas CCD detector, using graphite monochromated MoKα radiation. The data were treated using the CrysAlisPro software suite program package [66]. Analytical absorption corrections were applied to all data sets. All structures were solved using the dual-space algorithm of the SHELXT [67] program implemented in the Olex2 (version 1.5) crystallographic software [68]. Structure refinement was performed with SHELXL-2014 software [69]. The atom O4 in **2** was found to be disordered over two positions, O4A and O4B, with occupancies of 0.74(2) and 0.26(2), respectively. Details of the data collection and processing, structure solution and refinement are summarized in Table 2.

Crystallographic data for the structures reported can be obtained free of charge from the Cambridge Crystallographic Data Centre via www.ccdc.cam.ac.uk/data_request/cif quoting the deposition numbers CCDC 2209507 (**1**), 2209509 (**2**), 2209508 (**3**), and 2209510 (**4**).

### 3.5. Theoretical Calculations

The ORCA 5.0.3 package was used for all calculations [70,71,72]. Unless stated otherwise, the calculations were performed using ωB97X-D4 functional [73] with the minimally augmented ma-def2-TZVP basis sets [74,75]. The effective core potentials (ECP) included in the ma-def2-TZVP basis set were used to describe the core electrons of Cd and I atoms. Grimme’s atom-pairwise dispersion correction was employed [76]. The SCF and optimization convergence criteria were settled with *VeryTightSCF* and *TightOPT* keywords, respectively. Integration grids of high density (*Defgrid3* keyword) were used. The CCSD calculations were performed through the DLPNO-CCSD(T) scheme [77] using the def2-TZVPPD basis set [46,75] and the *TightPNO* keyword. For calculations of singlet molecules, the “*UseFullLMP2Guess false*” command was applied to adjust the energies of closed-shell and open-shell calculations. *AutoAux* keyword [78] was used to generate auxiliary basis sets in all cases. To obtain the binding energy (BE) of the X⋯Y assembly, the whole geometry was optimized at the DFT level, and individual electronic energies E_XY_, E_X_ and E_Y_ were calculated at the DLPNO-CCSD(T) level with no structural relaxation of the X and Y components. The final BE was calculated according to the equation BE_XY_ = E_XY_ − E_X_ − E_Y_. The counterpoise (CP) correction [49] was not applied unless stated otherwise. The interaction enthalpy Δ*H* was obtained from the vibrational energies calculated on the DFT-optimized structures of dimers and relaxed monomers, where the electronic energies were corrected at the DLPNO-CCSD(T) level for relaxed geometries of the monomers. The root mean square deviation (RMS) was defined as $RMS=\sqrt{\frac{1}{N}\sum_{i=1}^{N}{\left(E_{calcd.}-E_{fit.}\right)}^{2}}$, where *E*_calcd._ and *E*_fit._ are energies or enthalpies obtained from DFT/DLPNO-CCSD(T) calculations and linear regression, respectively. Visualization of molecular orbitals was made using Avogadro 1.2 program [79]. Analysis of bond critical points and non-covalent interactions indexes [36] was performed using Multiwfn 3.8 program [80]. Hirshfeld analysis and surface visualization were made using the CrystalExplorer 17.5 program [81]. Cartesian coordinates of the DFT-optimized structures are given in Listing S1.

### 3.6. Strains Culturing Conditions and Antibacterial Assay

Gram-negative bacteria (*Escherichia coli* ATCC 25922, *Pseudomonas aeruginosa* ATCC 27853, *Klebsiella pneumoniae* ATCC 700603, *Acinetobacter baumannii* ATCC BAA 747, *Pseudomonas aeruginosa* HUI PAM*β*L) and Gram-positive bacteria (*Staphylococcus aureus* ATCC 25923, *Staphylococcus aureus* HUI MRSA, *Staphylococcus aureus* ATCC MR 43300, *Staphylococcus haemolyticus* HUI MRCNS). All strains were stored as frozen stocks in 25 (*v*/*v*)% glycerol at −80 °C. *Pseudomonas aeruginosa* HUI PAM*β*L, *Staphylococcus aureus* HUI MRSA, *Staphylococcus haemolyticus* HUI MRCNS were obtained from Ukrainian hospitals. The Kirby–Bauer disc-diffusion assay was used to determine the antimicrobial susceptibility as the diameter (mm) of inhibition or minimum inhibitory concentration (MIC) value after incubation for two days and interpreted according to the European Committee on Antimicrobial Susceptibility Testing (EUCAST) guidelines [82]. Antibiotic susceptibilities of the studied strains are listed in Tables S10 and S11.

Antibacterial activity of **1**, **3** and **4** was studied by the MICs method using the broth microdilution approach. The compounds were dissolved in dmso:H_2_O = 1:1 mixture under heating in a water bath for 1 h to achieve a stock concentration of 1.25 µg/mL. Further, the resulting solutions were incubated at 37 °C for 12 h. Separately, the control of the growth in the experiments with sterile distilled water or solvent mixture without the addition of coordination compounds was carried out. If the solutions with high concentrations of dmso (50% and 25%) showed antibacterial activity, such an effect was not associated with the activity of the studied complexes and was neglected.

All bacteria were cultured in Cation-adjusted Mueller–Hinton broth (CAMHB) at 37 °C overnight. Then a sample of each culture was then diluted 40-fold in fresh broth and incubated at 37 °C for 1.5–3 h. The resultant mid-log phase cultures were diluted (CFU/mL measured by OD_600_) and added to each of the compound-containing plates, giving a cell density of 5 × 10^5^ CFU/mL and a total volume of 50 µL. All the plates were covered and incubated at 37 °C for 18 h without shaking. Growth inhibition of all bacteria was determined by measuring absorbance at 600 nm (OD_600_) using a Tecan M1000 Pro (Tecan Austria GmbH, Grödig, Austria) monochromator plate reader. The percentage of growth inhibition was calculated for each well using the negative control (media only) and positive control (bacteria without inhibitors) on the same plate as the references. The growth rates for bacteria had a variation of ±10%, which is within the reported normal distribution of bacterial growth.

The MIC was determined as the lowest concentration at which the growth was fully inhibited, defined by an inhibition ≥ 80%. In addition, the maximal percentage of growth inhibition is reported as DMax, indicating any compounds with partial activity.

## 4. Conclusions

Using the one-pot synthetic approach, we have prepared four new complexes, [Cu(HL^1^)(L^2^)Cl] (**1**), [Cu(HL^1^)(L^1^)]∙Cl∙2H_2_O (**2**), [Co(L^1^)_2_]∙Cl (**3**) and [Cd(HL^1^)I_2_]∙dmso (**4**) based on Schiff base ligand HL^1^ prepared in situ by condensation of benzhydrazide and 2-pyridinecarboxaldehyde. For **1**, under the experimental conditions, the oxidation of 2-pyridinecarboxaldehyde led to the formation of ligand HL^2^. According to the single crystal X-ray diffraction data, the crystal structures of **1**–**4** show broad supramolecular diversity of the complex architectures, including 1D polymeric chains in **1**, 2D polymeric layers in **2**, supramolecular trimers in **3** and 3D framework in **4**.

We evaluated the non-covalent interactions in structures **1**–**3** with special attention to the C–H⋯Cl contacts. After DFT and QTAIM analysis of a series of model {R_3_C–H}^0^⋯Cl^−^ assemblies, we propose the linear regressions Δ*H* = −678 × *ρ*(**r**_BCP_) + 3 and BE = −726 × *ρ*(**r**_BCP_) + 4 which coefficients differ from those proposed earlier for charged X–H⋯Y hydrogen bonds [10]. Although the *R*^2^ and MAD values of the present regression demonstrate that the dispersion of the data is not negligible, we believe these regressions will help in the assessment of energies of charged {C–H⋯Cl}^−^ contacts.

Complexes **1**, **3** and **4** were screened against some bacteria strains and were found to be active against nine Gram-negative and Gram-Positive bacteria. The MIC obtained for these compounds showed that the best activity was observed against *Acinetobacter baumannii* M*β*L, and **4** was the most effective against all the tested organisms.

## Figures and Tables

**Table 1:** Antibacterial activity of complexes **1**, **3** and **4**.

Strains	MIC, mg/L			
	1	3	4	Control
Gram-negative				
*Escherichia coli* ATCC 25922	312.5	312.5	312.5	>625
*Pseudomonas aeruginosa* ATCC 27853	312.5	312.5	312.5	>625
*Klebsiella pneumoniae* ATCC 700603	312.5	312.5	312.5	>625
*Acinetobacter baumannii* ATCC BAA 747	156.25	156.25	156.25	>625
*Pseudomonas aeruginosa* HUI PAM*β*L	312.5	625	312.5	>625
Gram-positive				
*Staphylococcus aureus* ATCC 25923	312.5	156.25	156.25	>625
*Staphylococcus aureus* HUI MRSA	312.5	312.5	312.5	>625
*Staphylococcus aureus* ATCC MR 43300	312.5	312.5	156.25	>625
*Staphylococcus haemolyticus* HUI MRCNS	312.5	312.5	156.25	>625

**Table 2:** Crystal data and structure refinement for **1**–**4**.

	1	2	3	4
Empirical formula	C_19_H_15_ClCuN_4_O_3_	C_26_H_25_ClCuN_6_O_4_	C_26_H_20_ClCoN_6_O_2_	C_15_H_17_CdI_2_N_3_O_2_S
Formula weight	446.34	584.51	542.86	669.58
Crystal system	triclinic	triclinic	trigonal	monoclinic
Space group	$P\;\bar{1}$	$P\;\bar{1}$	*R*-3	*P*2_1_/*c*
*a*/Å	8.5290(7)	8.4957(5)	26.3744(8)	8.1647(3)
*b*/Å	9.5833(8)	11.3628(5)	26.3744(8)	17.5032(5)
*c*/Å	12.4010(11)	14.9737(8)	20.1441(7)	14.1081(5)
*α*/°	76.236(8)	108.664(4)	90	90
*β*/°	81.807(7)	96.484(4)	90	91.205(3)
*γ*/°	76.962(7)	102.474(4)	120	90
*V*/Å^3^	954.89(15)	1311.00(12)	12,135.1(8)	2015.71(12)
*Z*	2	2	18	4
Calculated density/g cm^−3^	1.552	1.481	1.337	2.206
*T*, K	150.00(10)	160(16)	150.00(10)	150.00(10)
*μ*(Mo-K_α_)/mm^−1^	3.148	2.489	0.769	4.262
*F*(000)	454	602	5004	1256
Reflections collected/unique	5944/3588	8756/4946	14,741/5925	16,320/4531
*R*_int_	0.0301	0.0407	0.0298	0.0417
Reflections with *I* > 2*σ*(*I*)	3062	3794	4903	3789
*Θ*_min_, *Θ*_max/_°	3.686, 72.311	3.177, 72.249	2.567, 29.124	2.327, 28.989
*R*_1_, *I* > 2*σ*(*I*)	0.0372	0.0444	0.0363	0.0288
*wR*_2_ (all data)	0.1030	0.1213	0.0986	0.0600
*GoF*	1.038	1.012	1.042	1.060

## Data Availability

Crystallographic data for the structures reported can be obtained free of charge from the Cambridge Crystallographic Data Centre via www.ccdc.cam.ac.uk/data_request/cif quoting the deposition numbers CCDC 2209507 (**1**), 2209509 (**2**), 2209508 (**3**), and 2209510 (**4**).

## Supplementary Materials

The following supporting information can be downloaded at: https://www.mdpi.com/article/10.3390/molecules27238233/s1, Scheme S1: Schematic representation of the coordination compounds 1–4. Figure S1: IR spectrum of **1**; Figure S2: IR spectrum of **2**; Figure S3: IR spectrum of **3**; Figure S4: IR spectrum of **4**; Figure S5: IR spectrum of HL^1^; Figure S6: Dimeric structure of **1** optimized at the DFT level, Figure S7: DFT-optimized structure of the {H_2_L^1^·Cl}_0_ assembly, the plot of Laplacian of electron density, reduced density gradient plot; Figure S8: DFT-optimized model assemblies **1m**–**16m**; Figure S9: plot of Laplacian of electron density and reduced density gradient plot for **1m**; Figure S10: plot of Laplacian of electron density and reduced density gradient plot for **16m**; Figure S11: Plot of the BE vs. *ρ*(**r**_BCP_) dependence obtained for three reference assemblies from [10]; Figure S12: Plot of the Δ*H* vs. *ρ*(**r**_BCP_) data obtained using different basis sets; Figure S13: Plot of the Δ*H* vs. *ρ*(**r**_BCP_) data with and without DLPNO-CCSD(T) correction of the electronic energy; Table S1. Selected geometrical parameters for **1**; Table S2. Selected geometrical parameters for **2**; Table S3. Selected geometrical parameters for **3**; Table S4. Selected geometrical parameters for **4**; Table S5. DFT and DLPNO-CCSD(T)-corrected total energies and enthalpies of the model assemblies **1m**–**16m**; Table S6. Thermodynamic parameters of the model assemblies **1m**–**16m** and electron densities at the H⋯Cl bond critical points; Table S7. Binding energies of the model assemblies **1m**–**16m** calculated using different basis sets; Table S8. Geometry and binding energies of the selected model H-bonded assemblies from the S66 dataset calculated at different theory levels; Table S9. Selected properties of the {CH_4_⋯Cl}^−^ assembly optimized at various levels of theory. Table S10. The susceptibility of studied gram-positive strains to some known antibiotics. Table S11. The susceptibility of studied gram-negative strains to some known antibiotics. Listing S1. Cartesian coordinates (Å) of the monomers and dimers of the {R_3_C-H⋯Cl}^−^ and selected S66 model assemblies optimized at the ωB97X-D4/ma-def2-TZVP level.

## Sample Availability

Samples of the compounds are not available from the authors.
