# Supplementary material for: Supramolecular Diversity, Theoretical Investigation and Antibacterial Activity of Cu, Co and Cd Complexes Based on the Tridentate N,N,O-Schiff Base Ligand Formed In Situ
Source: Molecules. 2022 Nov 25;27(23):8233. doi: 10.3390/molecules27238233 (PMC9740120; doi:10.3390/molecules27238233)
Supplement: Supplementary file 1 [file molecules-27-08233-s001.zip › molecules-1970867-supplementary.pdf]

Electronic Supporting Information

Supramolecular diversity, theoretical investigation and antibacterial activity of Cu, Co and Cd complexes based on the tridentate N,N,O-Schiff base ligand formed *in situ*

Elena A. Buvaylo,[a] *Oksana V. Nesterova,*[b] *Evgeny A. Goreshnik,*[c] *Hanna V. Vyshniakova,*[d] *Svitlana R. Petrusenko,*[a] *and Dmytro S. Nesterov,*[b]*

[a] Department of Chemistry, Taras Shevchenko National University of Kyiv, Volodymyrska 64/13, Kyiv 01601, Ukraine

[b] Centro de Química Estrutural, Institute of Molecular Sciences, Instituto Superior Técnico, Universidade de Lisboa, 1049-001 Lisbon, Portugal; e-mail: dmytro.nesterov@tecnico.ulisboa.pt

[c] Department of Inorganic Chemistry and Technology, Josef Stefan Institute, Jamova 39 1000, Ljubljana, Slovenia

[d] L.V. Gromashevsky Institute of Epidemiology and Infectious Diseases NAMS of Ukraine, M. Amosova 5, Kyiv 03038, Ukraine

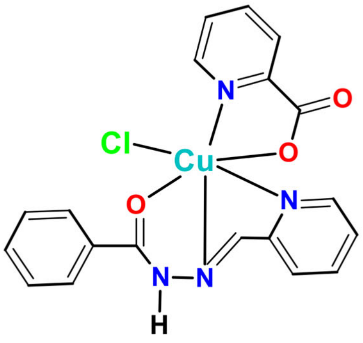

[Cu^{II}(HL^1)(L^2)Cl] **1**

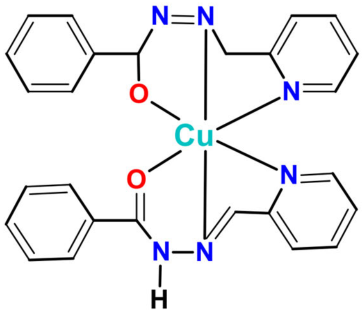

[Cu^{II}(HL^1)(L^1)]$^+$ **2**

uncoordinated Cl$^-$ counterion
is omitted for clarity.

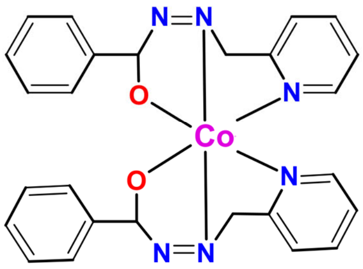

[Co^{III}(L^1)$_2$]$^+$ **3**

uncoordinated Cl$^-$ counterion
is omitted for clarity.

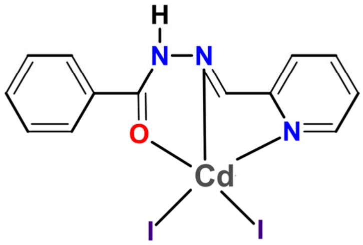

[Cd^{II}(HL^1)I$_2$] **4**

Scheme S1. Schematic representation of the coordination compounds **1**–**4**.

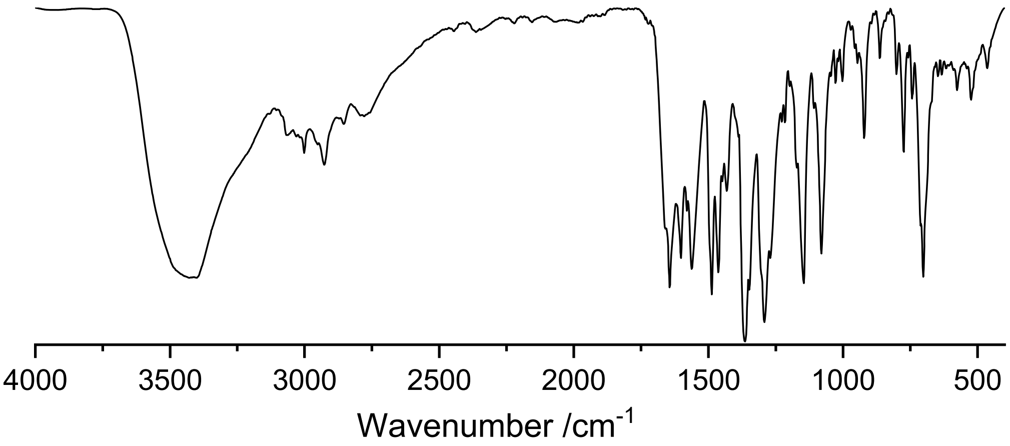

Figure S1. IR spectrum of **1**.

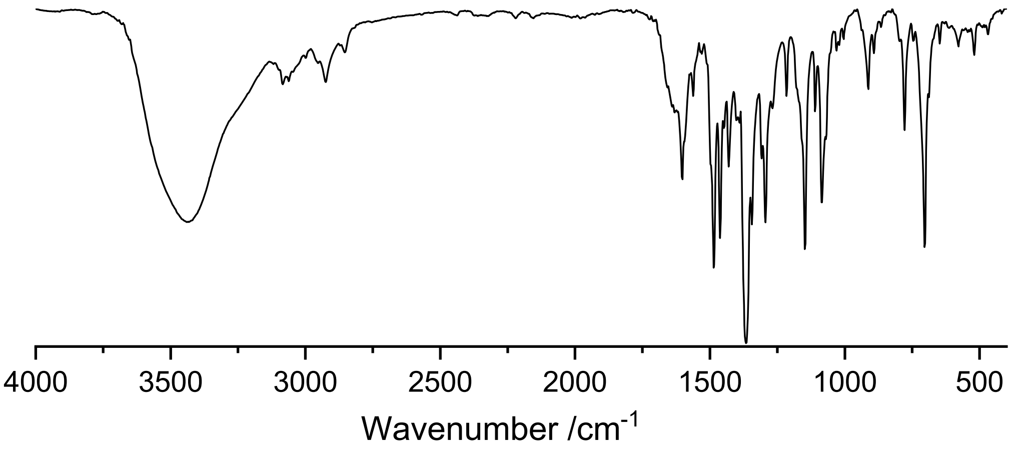

Figure S2. IR spectrum of **2**.

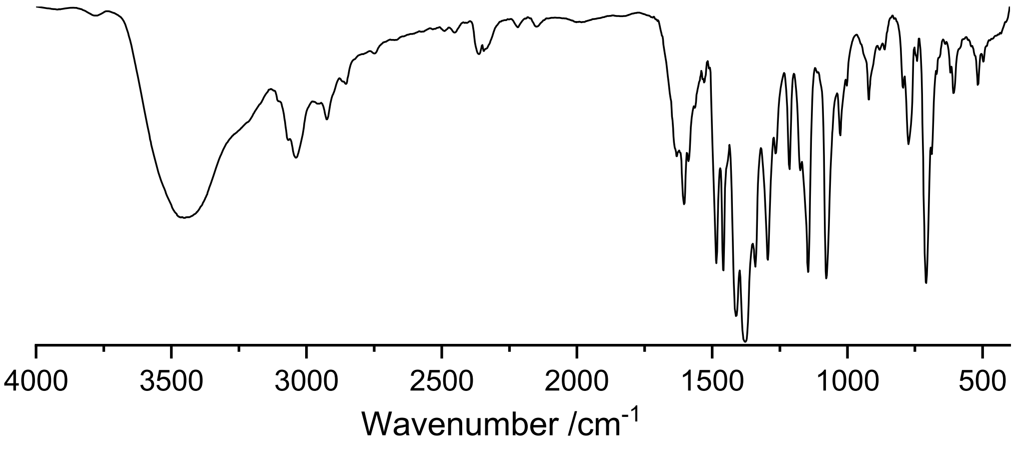

Figure S3. IR spectrum of **3**.

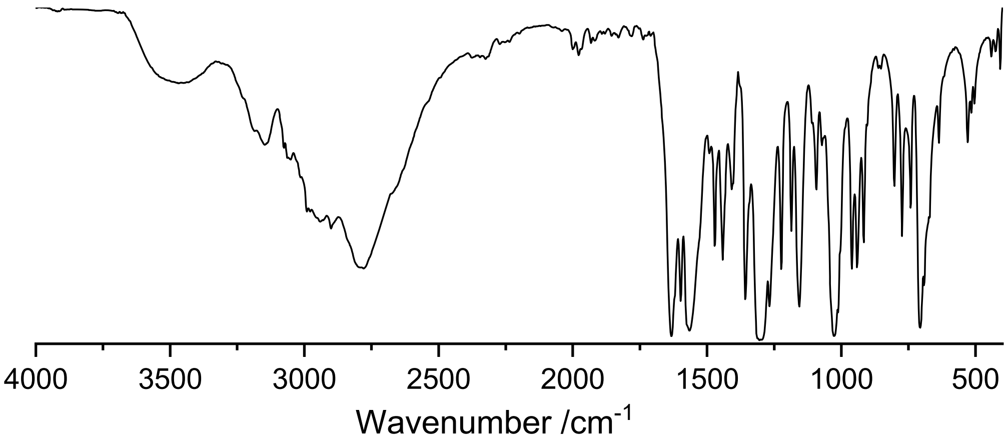

Figure S4. IR spectrum of **4**.

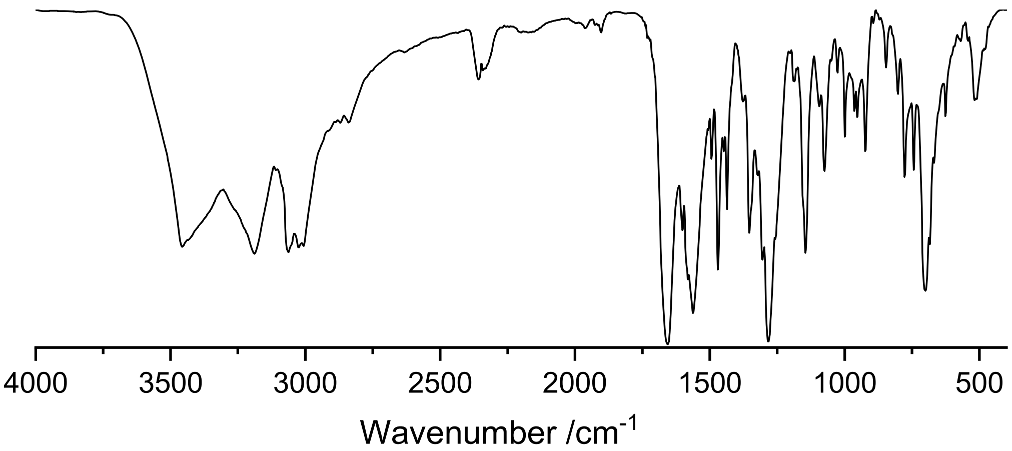

Figure S5. IR spectrum of HL^1.

Table S1. Selected geometrical parameters (distances/Å and angles/°) for **1**.

Cu1–O2	1.9766(19)	Cu1–N2	2.039(2)
Cu1–O3	2.500(2)	Cu1–N4	2.378(2)
Cu1–N1	2.020(2)	Cu1–Cl1	2.2591(7)
O2–Cu1–N1	81.59(8)	O3–Cu1–Cl1	89.12(5)
O2–Cu1–N2	88.07(8)	N1–Cu1–N2	168.14(9)
O2–Cu1–N4	88.02(8)	N1–Cu1–N4	99.29(8)
O2–Cu1–Cl1	173.00(6)	N1–Cu1–Cl1	95.73(6)
O2–Cu1–O3	86.09(7)	N2–Cu1–N4	74.41(8)
O3–Cu1–N1	114.01(7)	N2–Cu1–Cl1	95.19(6)
O3–Cu1–N2	70.78(7)	N4–Cu1–Cl1	98.82(6)
O3–Cu1–N4	144.84(7)		

Table S2. Selected geometrical parameters (distances/Å and angles/°) for **2**.

Cu1–O1	2.0149(19)	Cu1–N3	2.069(2)
Cu1–O2	2.582(2)	Cu1–N5	2.047(2)
Cu1–N2	1.931(2)	Cu1–N6	2.254(3)
O1–Cu1–N2	78.25(9)	O2–Cu1–N6	144.32(8)
O1–Cu1–N3	158.29(9)	N2–Cu1–N3	80.04(9)
O1–Cu1–N5	100.82(8)	N2–Cu1–N5	175.14(10)
O1–Cu1–N6	94.12(9)	N2–Cu1–N6	109.24(10)
O1–Cu1–O2	92.70(8)	N3–Cu1–N5	100.82(9)
O2–Cu1–N2	106.44(9)	N3–Cu1–N6	92.86(9)
O2–Cu1–N3	93.54(8)	N5–Cu1–N6	75.55(9)
O2–Cu1–N5	68.77(8)		

Table S3. Selected geometrical parameters (distances/Å and angles/°) for **3**.

Co1–O1	1.8927(13)	Co1–N3	1.9253(16)
Co1–O2	1.9099(14)	Co1–N5	1.8564(15)
Co1–N2	1.8566(15)	Co1–N6	1.9213(17)
O1–Co1–O2	91.01(6)	N2–Co1–N5	175.36(7)
O1–Co1–N3	165.19(6)	N2–Co1–N6	99.27(7)
O1–Co1–N6	89.87(6)	N5–Co1–O1	93.85(6)
O2–Co1–N3	92.90(6)	N5–Co1–O2	81.98(6)
O2–Co1–N6	165.31(6)	N5–Co1–N3	100.84(7)
N2–Co1–O1	82.35(6)	N5–Co1–N6	83.33(7)
N2–Co1–O2	95.38(6)	N6–Co1–N3	89.96(7)
N2–Co1–N3	83.06(7)		

Table S4. Selected geometrical parameters (distances/Å and angles/°) for **4**.

Cd1–O1	2.428(3)	Cd1–I1	2.7169(4)
Cd1–N2	2.314(3)	Cd1–I2	2.7209(4)
Cd1–N3	2.400(3)		
O1–Cd1–N2	66.86(9)	N2–Cd1–I1	118.76(8)
O1–Cd1–N3	135.73(9)	N2–Cd1–I2	119.50(8)
O1–Cd1–I1	97.98(6)	N3–Cd1–I1	103.17(7)
O1–Cd1–I2	99.52(7)	N3–Cd1–I2	101.70(8)
N2–Cd1–N3	68.87(10)	I1–Cd1–I2	121.490(12)

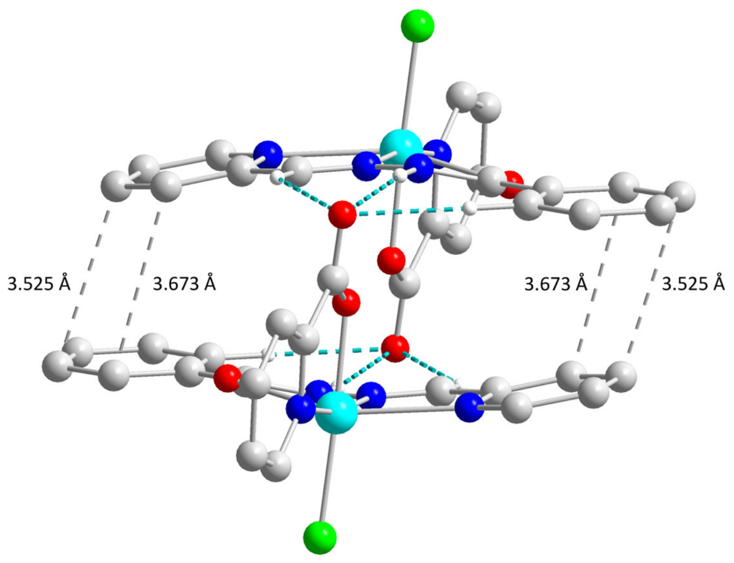

Figure S6. Dimeric structure of **1** optimized at the DFT level (ωB97X-D4 functional with the ma-def2-TZVP basis set for copper atoms and ma-def2-SVP basis set for all other atoms). Colour scheme: Cu, cyan; O, red; N, blue; C, grey; Cl, green; H, white.

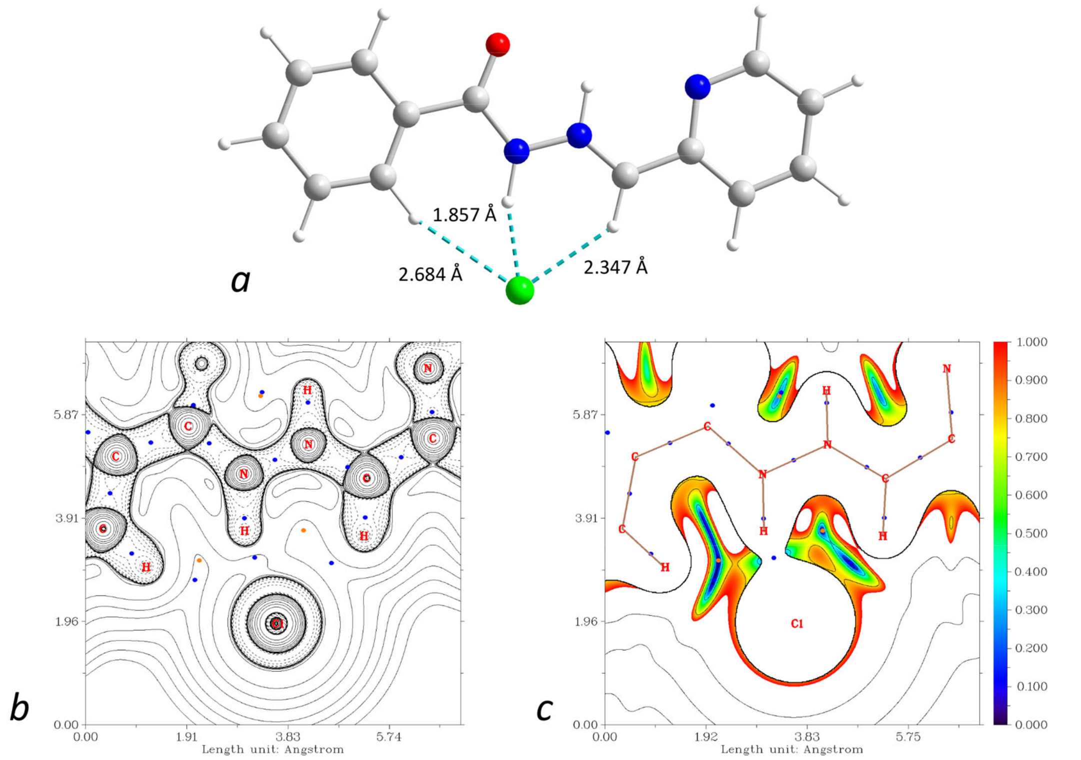

Figure S7. a: DFT optimized structure of the $\{H_2L^1{\cdot}Cl\}^0$ assembly. b: plot of the Laplacian of electron density $\nabla^2\rho(\mathbf{r})$ highlighting the Cl···H interactions (BCPs are shown as blue dots). c: plot of the reduced density gradient (RDG) at the same plane. Colour scheme: O, red; N, blue; C, grey; Cl, green; H, white.

Table S5. DFT and DLPNO-CCSD(T)-corrected total energies and enthalpies of the model assemblies **1m–16m** $\{R_3C–H\cdots Cl\}^-$ constructed from the substrates making a single C–H···Cl hydrogen bond with the chloride anion.[a]

Substrate[b]	*E*(el)[d]		*H*[e]		Model assembly[c]	*E*(el)[d]		Δ*H*[f]	
	CCSD	DFT	DFT	final[g]		CCSD	DFT	DFT	final[g]
CH_3 [h]	-39.762333	-39.874660	-39.840790	-39.728463	**1m** [h]	-499.562052	-500.170150	-500.133527	-499.525429
CH_4	-40.439312	-40.558072	-40.509342	-40.390583	**2m**	-500.237330	-500.851858	-500.800485	-500.185957
CHF_3	-337.873026	-338.427790	-338.397999	-337.843235	**3m**	-797.690884	-798.741497	-798.709343	-797.658730
$CHCl_3$	-1417.808116	-1419.387449	-1419.361710	-1417.782378	**4m**	-1877.629059	-1879.704147	-1879.676197	-1877.601108
C_6H_5 [h]	-231.121382	-231.747834	-231.654454	-231.028001	**5m** [h]	-690.929671	-692.052164	-691.957163	-690.834670
C_6H_6	-231.810060	-232.442416	-232.335979	-231.703623	**6m**	-691.615867	-692.744257	-692.636351	-691.507961
C_5H_6	-193.739971	-194.270318	-194.171727	-193.641381	**7m**	-653.545132	-654.571742	-654.470912	-653.444302
C_5H_5 [h]	-193.097508	-193.624790	-193.541380	-193.014098	**8m** [h]	-652.904487	-653.927932	-653.840423	-652.816978
C_6H_5F	-330.948884	-331.728219	-331.629059	-330.849725	**9m**	-790.758608	-792.033878	-791.933124	-790.657854
$C_5H_2F_4$	-590.265117	-591.383558	-591.312599	-590.194159	**10m**	-1050.083304	-1051.697331	-1051.624498	-1050.010470
C_6HF_5	-727.468961	-728.836049	-728.765763	-727.398675	**11m**	-1187.287827	-1189.150217	-1189.077809	-1187.215419
$C_6H_3F_2$ [h]	-429.392286	-430.312686	-430.233822	-429.313423	**12m** [h]	-889.202197	-890.618170	-890.537054	-889.121081
$C_6H_3Cl_2$ [h]	-1149.377786	-1150.979021	-1150.902062	-1149.300826	**13m** [h]	-1609.188054	-1611.284823	-1611.205588	-1609.108819
$C_6H_7F_5$	-731.163925	-732.555668	-732.413453	-731.021710	**14m**	-1190.972088	-1192.858825	-1192.714352	-1190.827614
$(CF_3)_3CH$	-1050.467648	-1052.226254	-1052.150714	-1050.392108	**15m**	-1510.295763	-1512.549149	-1512.473040	-1510.219653
$C_5H_4NC(O)H$ [j]	-360.9939352	-361.8691151	-361.7632467	-360.888067	**16m** [J]	-499.562052	-822.1864428	-822.0777254	-499.525429
Cl^-	-459.792982	-460.288676	-460.286316	-459.792982					

[a] Geometry optimization and thermodynamic calculations were performed at the ωB97X-D4/ma-def2-TZVP level for the gas phase standard conditions (P = 1 atm and T = 298.15 K). All geometries of dimers and monomers are relaxed. The electronic energy E(el) of dimers and monomers was corrected by the DLPNO-CCSD(T)/def2-TZVPPD single point calculations; [b] a set of aromatic and aliphatic compounds bearing C–H bonds; [c] the assembly where the chlorine anion forms a single C–H···Cl hydrogen bond with the model compound; [d] electronic energy (Hartree); [e] enthalpy at 298.15 K (Hartree); [f] interaction enthalpy, $\Delta H = H_{assembly} - H_{substrate} - H_{Cl-}$, at 298.15 K (Hartree); [g] $H_{final} = H_{DFT} - E(el)_{DFT} + E(el)_{CCSD}$; [h] radical species; [J] the assembly has two C–H···Cl contacts.

Table S6. Thermodynamic parameters of the model assemblies **1m–16m** and electron densities at the H···Cl bond critical points.[a]

Model	Δ*H*[b]		*p*(**r**), a.u.[d]	*d*(Cl···H), Å	∠(C–H···Cl), °
	DFT	final[c]			
**1m** [e]	-4.03	-3.98	0.011786	2.586	179.92
**2m**	-3.03	-2.98	0.009897	2.689	179.16
**3m**	-15.71	-15.61	0.024452	2.242	179.89
**4m**	-17.68	-17.64	0.032714	2.113	179.92
**5m** [e]	-10.29	-10.07	0.017639	2.396	174.52
**6m**	-8.82	-8.61	0.016063	2.441	174.49
**7m**	-8.08	-7.72	0.016576	2.437	159.37
**8m** [e]	-7.99	-7.69	0.017207	2.403	179.82
**9m**	-11.14	-10.99	0.017991	2.386	176.34
**10m**	-16.05	-16.12	0.027873	2.187	175.13
**11m**	-16.15	-16.39	0.027398	2.186	179.59
**12m** [e]	-10.61	-10.69	0.023492	2.256	178.26
**13m** [e]	-10.80	-10.90	0.024234	2.244	178.52
**14m**	-9.15	-9.59	0.024028	2.252	175.84
**15m**	-22.60	-23.17	0.038135	2.049	179.69
**16m**	-17.67	-17.58	0.020191[f]	2.337	166.44
			0.009298[g]	2.768	149.97

[a] Raw thermochemical data and details of the calculations are given in the Table S5; [b] interaction enthalpy at 298.15 K (kcal mol^{-1}); [c] enthalpy with *E*(el) corrected by the DLPNO-CCSD(T) calculations (Table S5 footnote) using relaxed structures of monomers; [d] electron density at the (3, –1) critical point of the H···Cl contact; [e] radical species; [f] Ar–H···Cl contact; [g] C(O)H···Cl contact.

Table S7. Binding energies of the model assemblies **1m–16m** calculated using different basis sets.[a]

	No extrapolation		Extrapolated basis sets					
	def2-TZVPPD		aug-cc-pV{D,T}Z		aug-ano-pV{D,T}Z		aug-ano-pV{T,Q}Z	
Model	–[b]	CP-corr.[c]	–	CP-corr.	–	CP-corr.	–	CP-corr.
**1m** [d]	-4.32	-3.67	-4.55	-4.18	-4.79	-4.23	-4.59	-4.13
**2m**	-3.19	-2.63	-3.29	-3.03	-3.57	-3.05	-3.38	-3.01
**3m**	-16.90	-14.74	-17.41	-15.47	-17.95	-16.10	-17.51	-15.88
**4m**	-18.18	-16.36	-18.60	-17.60	-19.16	-17.92	-18.76	-17.89
**5m** [d]	-9.67		-9.79					
**6m**	-8.13		-8.20					
**7m**	-7.90		-8.08					
**8m** [d]	-8.90		-9.02					
**9m**	-10.67		-10.81					
**10m**	-16.49		-16.96					
**11m**	-16.86		-17.28					
**12m** [d]	-10.99		-11.13					
**13m** [d]	-11.05		-11.40					
**14m**	-10.25		-10.16					
**15m**	-25.29		-24.59					
**16m**	-17.96		-18.18					

[a] Binding energies (kcal mol^{-1}) were calculated at the DLPNO-CCSD(T) level using unrelaxed geometries of monomers (full assemblies were optimized at the ωB97X-D4/ma-def2-TZVP level); [b] no counterpoise correction applied; [c] binding energies corrected for basis set superposition error (see the Experimental section for details); [d] radical species.

Table S8. Geometry and binding energies of the selected model H-bonded assemblies from the S66 dataset calculated at different theory levels.[a]

		Original S66 dataset data[b]		DFT optimized[c]	
Model	D–H···A	*d*(H···A), Å	∠(X–H···A), °	*d*(H···A), Å	∠(X–H···A), °
**01**	HOH···OH_2	1.963	173.71	1.964	173.32
**03**	HOH···NH_2CH_3	1.961	174.08	1.948	173.90
**04**	HOH···O=C(CH_3)$NHCH_3$	1.861	166.90	1.858	165.76
**09**	CH_3HNH···$OHCH_3$	2.200	161.98	2.227	159.16
**18**	HOH···NC_6H_5	1.954	176.98	1.936	177.41

			Basis set[d]							
			Normal		Extrapolated					
	Original S66 data[e]	Revised S66 data[f]	def2-TZVPPD		aug-cc-pV{D,T}Z		aug-ano-pV{D,T}Z		aug-ano-pV{T,Q}Z	
Model			–[g]	CP-corr.[h]	–	CP-corr	–	CP-corr	–	CP-corr.
**01**	-4.751	-4.982	-4.994	-4.505	-5.179	-4.890	-5.045	-4.877	-5.059	-4.889
**03**	-6.595	-6.896	-7.102	-6.349	-7.296	-6.899	-7.222	-6.869	-7.162	-6.818
**04**	-7.802	-8.183	-8.107	-7.210	-8.409	-7.879	-8.179	-7.877		
**09**	-2.777	-3.087	-3.182	-2.675	-3.227	-3.004	-2.963	-2.933		
**18**	-6.928	-6.927	-7.072	-6.208	-7.252	-6.781	-7.039	-6.736		
		**RMS**:[k]	0.125	0.657	0.274	0.161	0.166	0.182	0.196	0.086

[a] The S66 dataset belongs to the Benchmark Energy & Geometry Database [43] available at http://www.begdb.org. The model numbers correspond to the respective numbers in the dataset; [b] the atomic coordinates reported in the S66 dataset; [c] optimization at the ωB97X-D4/ma-def2-TZVP level; [d] all energies (kcal mol^{-1}) were calculate for the DFT optimized geometries; [e] binding energies as reported in the S66 dataset (CCSD/CBS + counterpoise correction level); [f] binding energies calculated at the MP2-F12/aV{T,Q}Z-F12 half-CP + [CCSD(F12*)–MP2-F12]/aVTZ-F12 half-CP + [CCSD(T)–CCSD]/haV{D,T}Z half-CP level (where CP means counterpoise correction) as reported in [45]; [g] no counterpoise correction applied; [h] binding energies corrected for basis set superposition error (see the Experimental section for details); [k] root mean square (RMS) deviation between the revised S66 binding energies and calculated herein at the indicated level.

Table S9. Selected properties of the $\{CH_4\cdots Cl\}^-$ assembly optimized at various levels of theory.

Method	Basis set	*d*(H···Cl)	*ρ*(**r**), a.u.[a]
ωB97X-D4	ma-def2-TZVP	**2.689**	**0.009897**
	ma-def2-QZVP	2.673	0.010674
	def2-TZVP	2.575	0.012729
	def2-TZVP-gCP [b]	2.591	0.012363
	def2-QZVPPD	2.672	0.010711
	aug-cc-pVTZ	2.669	
	aug-cc-pV6Z	2.673	
ωB97M-V	ma-def2-TZVP	2.691	0.010053
	ma-def2-QZVP	2.677	0.010815
	def2-QZVPPD	2.677	0.010826
	aug-cc-pVTZ	2.673	
	aug-cc-pV6Z	2.676	
M062X [c]	ma-def2-TZVP	2.697	0.009813
	ma-def2-QZVP	2.661	0.010784
	def2-QZVPPD	2.659	0.010820
	aug-cc-pVTZ	2.651	
	aug-cc-pV6Z	2.661	
SCS-MP2	ma-def2-TZVP	2.794	
	ma-def2-QZVP	2.711	
	def2-QZVPPD	2.713	
	aug-cc-pVTZ	2.598	
	aug-cc-pV6Z	2.603	
DLPNO-CCSD(T)	ma-def2-TZVP	2.743	
	def2-TZVPPD	2.648	
	def2-QZVPPD	2.651	
	aug-cc-pVTZ	2.617	

[a] electron density at the (3, –1) critical point of the H···Cl contact; [b] geometrical semi-empirical counterpoise correction (gCP) as described by Kruse and Grimme [56]; [c] *D3Zero* keyword was used for dispersion correction.

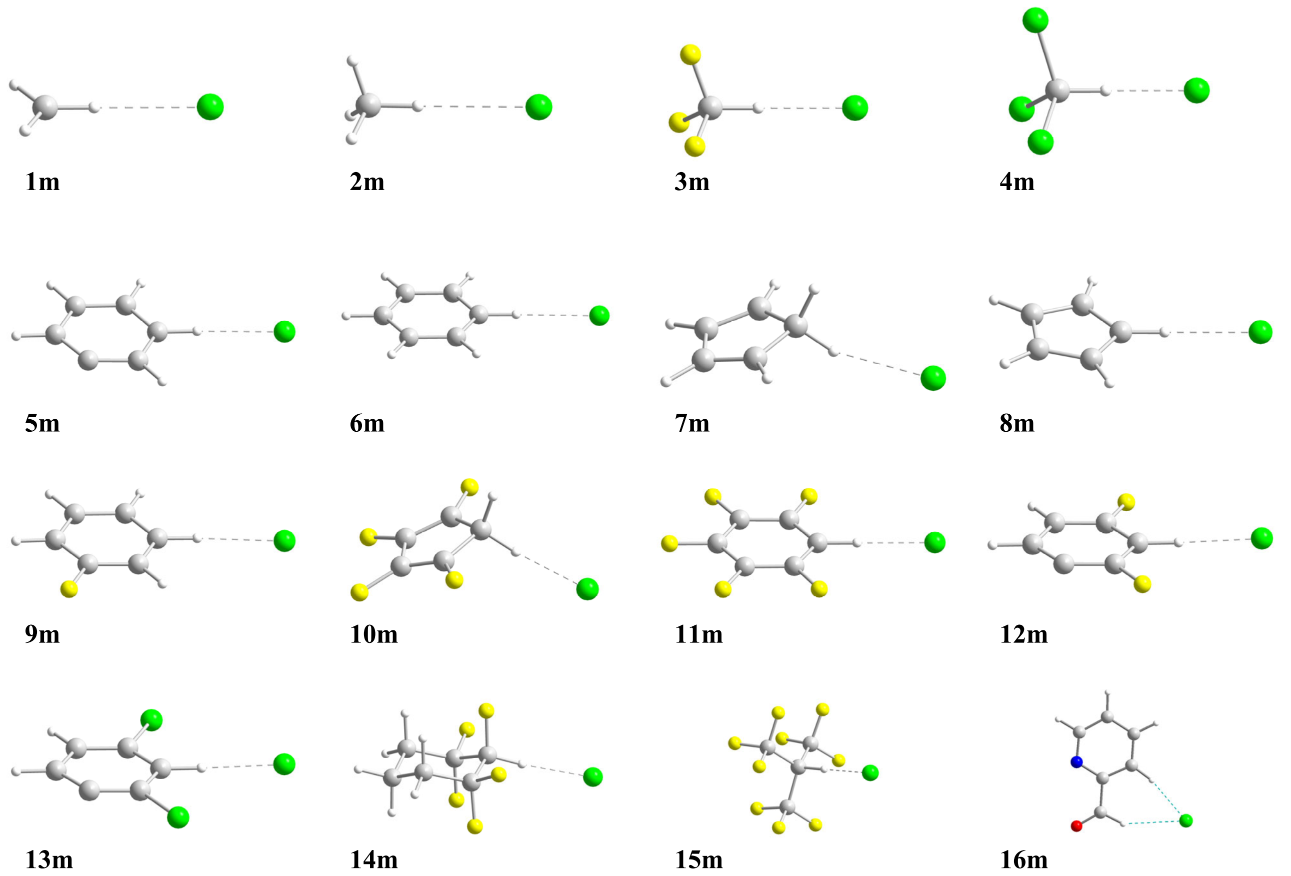

Figure S8. Model assemblies **1m-16m** optimized at the ωB97X-D4/ma-def2-TZVP level. Colour scheme: C, grey; H, white; O, red; N, blue; F, yellow; Cl, green.

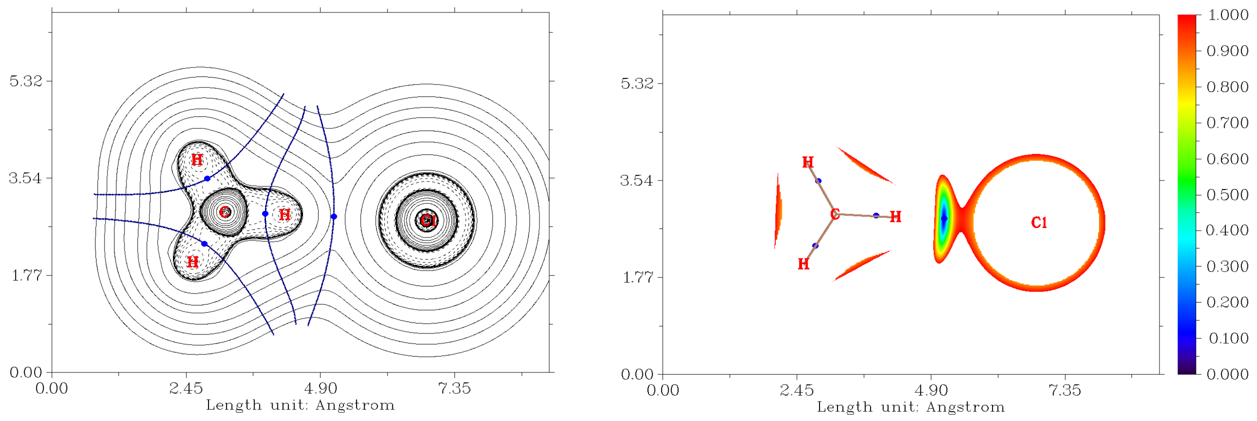

Figure S9. Left: diagram of the Laplacian of electron density, $\nabla^2\rho(\mathbf{r})$, showing all bond critical points (blue dots) in the structure **1m**. Right: the RGD isosurface of the same projection.

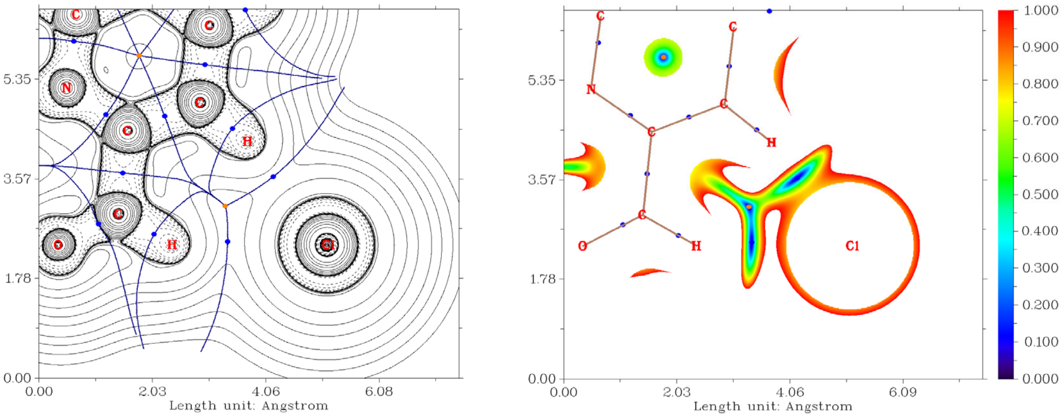

Figure S10. Left: diagram of the Laplacian of electron density, $\nabla^2\rho(\mathbf{r})$, showing selected bond critical points (blue dots) in the structure **16m**. Right: the RGD isosurface of the same projection.

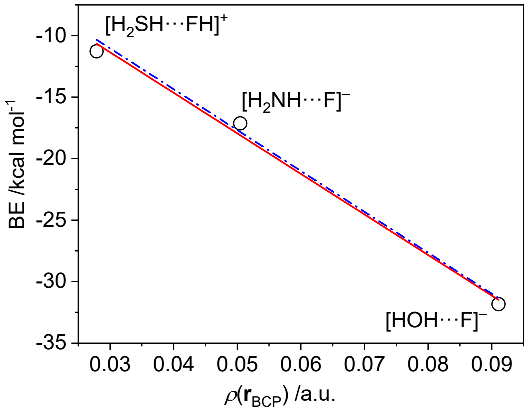

Figure S11. Plot of the BE *vs*. $\rho(\mathbf{r}_{\mathrm{BCP}})$ dependence obtained for three reference assemblies [10] at the full geometry optimization at the ωB97X-D4/ma-def2-TZVP level with subsequent extraction of electronic energies at the DLPNO-CCSD(T)/def2-TZVPPD level using non-relaxed structures of monomers and *UseFullLMP2Guess* option set to "true". Solid red line represents a linear fit with the slope of –329.7 and dash-dot blue line was bult using the dependence by Emamian and Lu for charged complexes (slope of –332.34) [10].

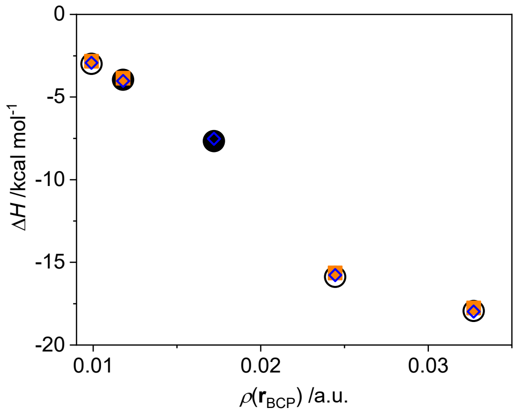

Figure S12. Plot of the ΔH *vs*. $\rho(\mathbf{r}_{BCP})$ data obtained at the ωB97X-D4/ma-def2-TZVP // DLPNO-CCSD(T) level, showing a negligible variation of ΔH depending on the basis set used in the DLPNO-CCSD(T) calculations: def2-TZVPPD (empty and filled circles, closed-shell and open-shell models, respectively), ma-def2-QZVPP (filled orange squares) and aug-cc-pVQZ (blue rhombs).

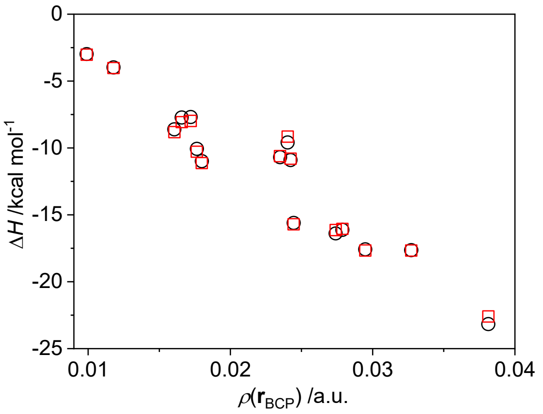

Figure S13. Plot of the ΔH *vs*. $\rho(\mathbf{r}_{BCP})$ data with (black circles) and without (red squares) DLPNO-CCSD(T) correction of the electronic energy.

Table S10. The susceptibility of studied gram-positive strains to some known antibiotics.

Strain	penicillin	oxacillin	erythromycin	azithromycin	clindamycin	chloramphenicol	ciprofloxacin	moxifloxacin	vancomycin	teicoplanin	gentamicin	linezolid
Staphylococcus aureus ATCC 25923	**R**	**S**	**S**	**S**	**S**	**S**	**S**	**S**	**S**	**S**	**S**	**S**
Staphylococcus aureus HUI MRSA	**R**	**R**	**R**	**R**	**R**	**R**	**R**	**S**	**R**	**S**	**R**	**R**
Staphylococcus aureus ATCC MR 43300	**R**	**R**	**R**	**R**	**R**	**S**	**S**	**S**	**S**	**S**	**R**	**S**
Staphylococcus haemolyticus HUI MRCNS	**R**	**R**	**R**	**R**	**R**	**R**	**R**	**S**	**R**	**S**	**R**	**S**

*R – resistance; *S – susceptibility.

Table S11. The susceptibility of studied gram-negative strains to some known antibiotics.

Strain	amikacin	ceftazidime	ciprofloxacin	colistin	gentamycin	imipenem	levofloxacin	meropenem	piperacillin-tazobactam	tobramycin
Escherichia coli ATCC 25922	**S**	**S**	**S**	**S**	**S**	**S**	**S**	**S**	**S**	**S**
Pseudomonas aeruginosa ATCC 27853	**S**	**S**	**S**	**S**	**S**	**S**	**S**	**S**	**S**	**S**
Klebsiella pneumoniae ATCC 700603	**S**	**S**	**S**	**S**	**S**	**S**	**S**	–	–	**S**
Acinetobacter baumannii ATCC BAA 747	**S**	**IR**	–	–	–	**IR**	–	–	–	–
Pseudomonas aeruginosa HUI PAM*β*L	**S**	**S**	**R**	**S**	**S**	**R**	**R**	**R**	**S**	**S**

*R – resistance; *IR – intermediate resistance; *S – susceptibility.

Listing S1. Cartesian coordinates (Å) of the monomers and dimers of the $\{R_3C\text{-}H\cdots Cl\}^-$ and selected S66 model assemblies optimized at the ωB97X-D4/ma-def2-TZVP level.

CH_3 doublet
```
C -1.97380507947810 4.32186286902612 8.96576470668920
H -1.01409839840022 4.19451148667495 8.48460538717273
H -2.04756087792371 4.88941688782189 9.88294256611418
H -2.85977191061179 3.88182324926331 8.52963941421141
```

CH_4 singlet
```
C -2.07878297286592 4.57276406296107 8.83884660425409
H -1.09984131830929 4.33832304526910 8.41874653374154
H -2.46059317247163 5.48716918810762 8.38291195370470
H -1.98748100242960 4.71453508222231 9.91650728705564
H -2.76721128814759 3.75105724599661 8.63723512855354
```

CHF_3 singlet
```
C -1.90705617646819 4.53712209224883 8.76404906167646
H -0.92338550857607 4.31102676999187 8.34633334137628
F -2.40203892646718 5.65523523920816 8.21875594901643
F -1.82423396741380 4.71270139704984 10.08873107604169
F -2.76020191015218 3.53417195286865 8.52207632820778
```

$CHCl_3$ singlet
```
C -1.86562792207264 4.52757598061760 8.74647244542898
H -0.88932254960803 4.30339292791726 8.33190191291299
Cl -2.45158097229949 6.00225620740794 7.98720738842310
Cl -1.68069426532936 4.74471783307601 10.48234857292274
Cl -2.92969077976788 3.17231450234854 8.39201543663079
```

C_6H_5 doublet
```
C 0.26840535581976 6.71469559552830 3.16495258559374
C -0.86859483907816 6.67670535558871 3.93041843820006
C -2.02484517594994 7.37512072413441 3.69474345760983
H -2.89195833200258 7.30011147791141 4.34175774083043
C -2.03348861963038 8.19900791277065 2.56675864988049
H -2.92182758858451 8.77600162910876 2.32999685277201
C -0.91044855532547 8.28051471510754 1.75029027413828
H -0.92733407933579 8.92304502673290 0.87678321768511
C 0.23379344338561 7.54599346387210 2.04294263212007
H 1.10511671695502 7.61623164058126 1.39952218849826
H 1.15266371837969 6.13545049631803 3.40722310859088
```

C_6H_6 singlet
```
C 0.25667914524893 6.75677061584102 3.17483961942072
C -0.85936910377995 6.67887482447875 3.99940157342419
H -0.83672911829798 6.05239136788199 4.88488299019983
C -2.00409049548910 7.40358606574746 3.68964987891667
H -2.87528213093902 7.34287132597947 4.33339421453271
C -2.03270140864077 8.20605975988943 2.55528982222698
H -2.92634005504384 8.77176386120302 2.31346691833244
C -0.91664249053557 8.28395826649110 1.73071589085262
H -0.93935316649602 8.91045534938105 0.84524704837631
C 0.22806327785317 7.55925332882851 2.04047289422297
H 1.09927282262197 7.61995538524040 1.39674972042808
H 1.15032711006587 6.19107417068212 3.41664328037184
```

C_5H_6 singlet
```
C -0.12266009154560 6.85622164812623 3.32812858918990
C -1.34557022534672 7.24817247553813 3.71223158074959
C -1.91566522722609 8.12913893696221 2.68152628399659
H -2.89484701905830 8.58879986151408 2.73698164682421
C -1.03354524886694 8.26380948855340 1.68131452359006
H -1.16255582263637 8.84890340868618 0.78001797589323
C 0.19891545836943 7.46487971469110 1.99360288106758
H 1.09519150791224 8.09605983192952 2.03739501501879
H 0.54820785868848 6.20479110481789 3.87299294403135
H -1.84740646093698 6.96954026247223 4.63075161952472
H 0.39381527064686 6.69976326670896 1.23182694011398
```

C_5H_5 doublet
```
C -0.04146981182253 6.90483014004255 3.29925786129387
C -1.32227517607939 7.19498429145291 3.66791676488179
C -1.90400064990828 8.09886810859716 2.65213091730974
H -2.90996172934393 8.49641165327879 2.68147411266109
C -0.96023909286276 8.33085704475122 1.69713481192819
H -1.06282862709352 8.94798259039575 0.81623118672798
C 0.21412043823072 7.59857264380156 2.07349199711613
H 1.14435946433775 7.57464765898375 1.51901214083368
H 0.65965110009593 6.27208405485581 3.82398719110481
H -1.84155485547872 6.83668805316598 4.54707943830207
```

C_6H_5F singlet
```
C 0.26773253274618 6.76615606268485 3.16116206295249
C -0.86017606011008 6.70991589473476 3.95877314710108
F -0.83630078943296 5.93221473392237 5.06177818818838
C -2.01147280832804 7.41921896102231 3.67040351419719
H -2.86920832214270 7.34163964108196 4.32783049075501
C -2.02675152423509 8.21653249922289 2.53272146220345
H -2.92050433260313 8.78058289572412 2.28922986193891
C -0.90870050436481 8.29375325210296 1.71072740306579
H -0.92805006459752 8.91771254196649 0.82449257439583
C 0.23390730942194 7.56881276481704 2.02767120477367
H 1.10857988423150 7.62614513658449 1.38901860353991
H 1.14573331220382 6.19132595814068 3.43057684483297
```

$C_5H_2F_4$ singlet
```
C -0.09645481210754 6.88667777174782 3.25890056488900
C -1.28400941008053 7.28415443314944 3.71079296152428
C -1.92722814271310 8.11968984520930 2.67820770987776
F -3.12488865393773 8.66078911354995 2.85265889257603
C -1.11240595586403 8.20406715704606 1.62872742647524
F -1.31242039511242 8.86033419858236 0.48714751982538
C 0.14586969919799 7.42857367513774 1.88178303031541
H 1.02862402409862 8.07502399816230 1.85409062573582
F 0.80103012550090 6.12150228548619 3.87753962036955
F -1.86732388866509 7.02277703127247 4.87244755528323
H 0.27784432783803 6.62524252087685 1.15032100029419
```

C_6HF_5 singlet
```
C 0.26822499800053 6.72045309259008 3.16859631969563
C -0.85322381637110 6.62886675868306 3.97842747340870
C -1.98081852900987 7.37223254037058 3.65991695163184
F -3.06325918742216 7.29285199457432 4.42732082306474
C -1.98353492531865 8.19757479764267 2.54433677922008
F -3.06902315598591 8.90928066878121 2.24638060058289
C -0.84766478452263 8.26832505764156 1.75265405940276
F -0.86336546152231 9.06494987018705 0.68009201612178
C 0.28540233750989 7.53592421249874 2.05272751756382
H 1.16638316343412 7.59982468865976 1.42753155383550
F 1.34713571891103 5.99898942438362 3.48569794509437
F -0.85877609732791 5.84024301257738 5.05162692200971
```

$C_6H_3F_2$ doublet
```
C 0.19184069028969 6.71753207610953 3.18790288535724
C -0.92834369668365 6.66331302536734 3.97597705504737
C -2.07261893559044 7.37378876413448 3.73394176513786
H -2.93751975186421 7.30130261193007 4.38396978050928
C -2.09549795640558 8.20491623387504 2.61178216828000
H -2.96716470453683 8.79569641812703 2.35846523651731
C -0.96624791409299 8.26250238034830 1.81367220203299
F -0.98234714928567 9.06122585211840 0.72972844821467
C 0.18752177086255 7.53828964609052 2.06966748426611
H 1.05228039688343 7.61002452343405 1.42133905039611
F 1.28506385263126 5.99678396439565 3.47353416976691
```

$C_6H_3Cl_2$ doublet
```
C 0.16941508829385 6.70962360187008 3.21272471600905
C -0.95635986269473 6.66583391116425 3.98966309512282
C -2.10379896006884 7.37383228921824 3.75301302599254
H -2.96923300481159 7.30238895863183 4.40213288307785
C -2.11966738511554 8.20229267949276 2.63084373010784
H -2.99640089507874 8.78964915000614 2.38684443384683
C -0.99267845844717 8.26836378803842 1.82234724579016
Cl -1.01348540611213 9.29700270072338 0.42943729798067
C 0.15480872928448 7.53539426851772 2.09265473123850
H 1.02183171578092 7.60266112574741 1.44757154675424
Cl 1.57253504117705 5.77833302252014 3.59274753960534
```

$C_6H_7F_5$ singlet
```
C 0.23508639514295 6.47991187219110 3.12831786155003
C -0.67845031604236 7.04084060356477 4.19535583021533
C -2.11721323035063 7.25669861729713 3.70965375497932
F -2.66318359284148 6.01298737067804 3.46114872614296
C -2.15250257725769 8.04697726664729 2.39590469739440
F -3.44074987459560 8.07472450215542 1.95009818902457
C -1.23563371248630 7.48245598664823 1.33370948825731
H -1.63428168122187 6.50791582625050 1.04009149688821
C 0.19234704277514 7.34615265684198 1.86720496898588
H 0.60039869187969 8.33628044466736 2.08554911732289
H 1.24237778041383 6.42417049282210 3.54597822076286
H -2.70781028888128 7.76339896435106 4.47656887687979
F -0.71451159754808 6.21286230572122 5.27803780073570
F -0.20082636094972 8.24179069181009 4.64169220192893
H -0.10036170299089 5.46257200985382 2.91079529105583
H 0.82632685625868 6.90013060558336 1.09848430057954
F -1.81021110704709 9.34094123241566 2.67587096163011
H -1.28131371683477 8.14408296044936 0.46630057364749
```

$(CF_3)_3CH$ singlet
C 0.12798654953307 -0.19243770539610 -0.06491428203301
C 1.66731801187910 -0.21556008916195 -0.12585051589074
H -0.22590111510461 -0.09715143110187 -1.09285973410965
C -0.40363185110813 1.03292471855314 0.70330226733335
C -0.44843144438840 -1.51185276942927 0.48419963544426
F -0.26794482340540 2.13730673374078 -0.04202970912977
F 0.24511391172586 1.22578930037049 1.85483523623670
F -1.70570451459275 0.89439916616012 0.97922789085877
F 0.22765774524057 -2.56442032588149 0.00951224612987
F -1.72425095653072 -1.64939499088392 0.10078155887287
F -0.40699069712862 -1.56190742693062 1.81836222238477
F 2.15622266304062 1.01243852716428 -0.33323134736137
F 2.07431146081850 -0.98523148862128 -1.14357053416346
F 2.20827970748550 -0.69191791556747 0.99870295081395

C5H4NC(O)H singlet
H 9.42272315483127 12.89286638745433 8.24884332444205
H 9.16216104834353 12.91591859934731 10.57265560897453
C 9.39572146381304 13.99795123121022 8.13621520761986
C 9.11788706720193 13.99940587179739 10.60444952740904
O 9.49948640131372 14.51336091159344 7.05794517796147
C 9.22230077071459 14.73558070729360 9.43164374481405
C 8.95704708825973 14.67906611100281 11.80570557894785
N 9.17659605133062 16.06867369794230 9.39240240623532
H 8.87202232223898 14.13912627189535 12.74235574767959
C 8.90842570674471 16.06122304407181 11.77466931521113
C 9.02290463226635 16.70642253485517 10.54280375499479
H 8.78430349309157 16.64092122012973 12.68205438914224
H 8.98809079984990 17.79154341140645 10.48878621656804

1m: CH_3···Cl doublet
C -1.96909594321890 4.32126864706983 8.96341957753402
Cl 1.29157389254517 3.88774542255138 7.32466327807708
H -1.00242079238409 4.19292919513097 8.47871214288010
H -2.05800041847670 4.88775664555904 9.88337917290991
H -2.86571911233413 3.88566000502643 8.53744118086350

2m: CH_4···Cl singlet
C -2.07617867198943 4.57195570321873 8.83771254694709
Cl 1.32705219723269 3.79954078394899 7.37720525557999
H -1.09387379924355 4.33731519375182 8.41617960635786
H -2.46319518312269 5.48802949316804 8.38383741062487
H -1.99017741354705 4.71517965777270 9.91805974653246
H -2.77048468632131 3.75136857664543 8.63845819684723

3m: CHF_3···Cl singlet
C -1.88695463897459 4.53243613820426 8.75555279137992
Cl 1.12503461522703 3.83732179388886 7.47925612581011
H -0.89410409767181 4.30448585847730 8.33401680290932
F -2.41844929372581 5.65802763632426 8.22619532912095
F -1.84096107822339 4.71634013477968 10.09482405900683
F -2.77644738048181 3.53896768358185 8.52935677390163

4m: $CHCl_3$···Cl singlet
C -1.83962111909896 4.52131925832057 8.73536244044556
Cl 1.06286681348319 3.85243994255110 7.50573244006485
H -0.84076503138223 4.29164691533689 8.31135433911479
Cl -2.45503884064151 6.00128289923430 7.99004388478810
Cl -1.68567648078167 4.74550533131544 10.48190288011543
Cl -2.93364721542919 3.17538489849789 8.39480589759996

5m: C_6H_5···Cl doublet
C 0.26345900559179 6.71748533976698 3.16546790431808
C -0.87307810086773 6.67613182406107 3.93274621439970
C -2.03168238056827 7.37404962317967 3.69798983881197
H -2.90045447229589 7.29901615125524 4.34431291404458
C -2.03695202048297 8.19871502433942 2.56854234780373
H -2.92660324421185 8.77647683325626 2.33119743048271
C -0.91134774723834 8.27861620166694 1.75517876132113
H -0.92082876754536 8.92046415980973 0.88028275110366
C 0.23933440031557 7.54762879974039 2.03958205526024
H 1.12426873743796 7.61152366624567 1.40007469240245
Cl 3.18378430789679 7.67306479843009 0.17733094991754
H 1.15597028196829 6.14552757824847 3.39558414013419

6m: C_6H_6···Cl singlet
C 0.25226433147001 6.76032748044143 3.17344772981190
C -0.86313550747053 6.67953864537968 4.00020171731388
H -0.83971450870860 6.05196346503049 4.88681046308461
C -2.01113841342641 7.40257175674226 3.69352369404628
H -2.88362024008693 7.34127551519589 4.33773630986730
C -2.03562250228974 8.20493750391649 2.55801888894910
H -2.93021262531310 8.77161001620511 2.31490428511193
C -0.91630812219108 8.28129926581714 1.73543021690887
H -0.93316080234079 8.90752253016326 0.84887314474130
C 0.23547379094246 7.56108688605415 2.03556506837672
H 1.11716732271640 7.61762096294081 1.39417344510110
Cl 3.21316734457226 7.66776082358792 0.14369947917790
H 1.15136993212606 6.19934514852527 3.40767555750907

7m: C_5H_6···Cl singlet
C -0.13265762940804 6.91854381973704 3.31734879978166
C -1.37343861112010 7.23419985418393 3.72776908756069
C -2.02061874535964 8.05308690287287 2.69404065227556
H -3.02245482113585 8.46245290279528 2.76559096765987
C -1.16200985335853 8.22117352315267 1.67319294977362
H -1.33597298592847 8.79140861698908 0.76945627466788
C 0.12398560312843 7.52490830296688 1.97666879800378
H 0.99817098892413 8.20446658682142 1.96710830668929
Cl 3.16351167511281 9.12584524624340 1.33516351825132
H 0.60115310244448 6.33962433142218 3.86373573849909
H -1.82990184894702 6.95244587877781 4.67044757963294
H 0.37826312564781 6.77117403403730 1.22040732720424

8m: C_5H_5···Cl doublet
C -0.04203444750613 6.91553749896362 3.28397883942996
C -1.32088458213747 7.19366916447773 3.66823783004108
C -1.91536395246982 8.09598287735986 2.66266661543523
H -2.92464244259103 8.48847746436700 2.70050132937170
C -0.97747521712324 8.33529541108011 1.70208823267649
H -1.08501432068048 8.95423450311269 0.82234326271532
C 0.21423641298662 7.61261728246825 2.05399755074621
H 1.14814095761207 7.59678770046810 1.48714601591991
Cl 3.20560235325054 7.56380881249005 0.24697481439942
H 0.67267303115465 6.28616636575996 3.79552066335949
H -1.83012779249570 6.82775291945239 4.55194484590514

9m: C_6H_5F···Cl singlet
C 0.26674311616670 6.77470372622889 3.16872700355386
C -0.86533649812277 6.71078611020754 3.95785188378738
F -0.85134568405371 5.92433917618468 5.06940598858337
C -2.02054252256880 7.41302486615574 3.66954551564626
H -2.88212119613989 7.32855345068881 4.32211477752799
C -2.02859491102743 8.21425614293853 2.53185884117097
H -2.92424686312581 8.77584219841724 2.28327416441872
C -0.90316637433357 8.29615061905574 1.72074713934627
H -0.91210749865293 8.92096304541159 0.83417997574209
C 0.24835234205218 7.57899432930333 2.03390539959093
H 1.13684917416214 7.63880044145065 1.40011456381840
Cl 3.14056211717557 7.66364211213995 0.10561720956272
H 1.15148479846833 6.20680378181722 3.43271753725104

10m: $C_5H_2F_4$···Cl singlet
C -0.10435304289611 6.87502441367422 3.24316466860186
C -1.28501248811679 7.28629935768283 3.70768473844182
C -1.92532053448408 8.11689120173782 2.68011992028231
F -3.12461799154952 8.68494579282323 2.86059960245404
C -1.11368200187082 8.18330835992639 1.62386732865164
F -1.34168967462497 8.83251386293579 0.47990814506489
C 0.14731077367242 7.42968856966928 1.88174984023360
H 1.04012649924046 8.10563413233749 1.87198800258838
Cl 2.85939308184493 9.31049796977934 1.72831309283407
F 0.77318496244038 6.08993424387518 3.87188732595652
F -1.85932086733476 7.03979684805204 4.89230605406899
H 0.32201128367887 6.64479524750625 1.13934128082183

11m: C_6HF_5···Cl singlet
C 0.26711358383385 6.72569501029794 3.16175959287655
C -0.85472938207722 6.63218262603847 3.97378428468590
C -1.98270059306236 7.37204184115730 3.66123128892809
F -3.07440585865024 7.29244109534313 4.43557951290390
C -1.97947319222922 8.19405572417334 2.54688429487953
F -3.07424863505465 8.91074953298160 2.24951374302359
C -0.84127924723955 8.26256982735446 1.75539873263903
F -0.88383290447845 9.06744569401957 0.68722421351707
C 0.29912824479371 7.53671857852895 2.04297719134706
H 1.20136930075997 7.60366167273503 1.40262817553975
Cl 2.97262973962497 7.73022388140986 0.12777103836813
F 1.33416727609735 5.99364949913788 3.50260567984883
F -0.86362833681816 5.83830501682240 5.05572225144253

12m: $C_6H_3F_2$···Cl doublet
C 0.18953801772978 6.72253272712193 3.18100745660767
C -0.93132775422386 6.66505954630091 3.97437476094001
C -2.07815319521040 7.37330488564404 3.73847770668458
H -2.94330543348289 7.30176214644363 4.38910322853074
C -2.09437351159762 8.20233355255391 2.61454666229875
H -2.96401205131850 8.79596510887809 2.35740906903050
C -0.96153216886653 8.25693552622110 1.81674204311873
F -1.00591182609659 9.06371939829490 0.73745374688769
C 0.20035594223824 7.53920793278492 2.05951401201386
H 1.08455095159601 7.61437864168326 1.40200152326086
Cl 2.90656339779247 7.82765450406949 0.08877975447410
F 1.27113763153990 5.99017603000373 3.48935003615247

13m: $C_6H_3Cl_2$···Cl doublet
C 0.17124721033634 6.71819519891343 3.20281755831644
C -0.95275563234694 6.66706878650876 3.98467816898089
C -2.10420604901631 7.37105786293945 3.75383788236502
H -2.97005489100678 7.29739511450828 4.40302487009250
C -2.11683849782567 8.19978962619159 2.63137997989966
H -2.99322892173609 8.78783290576534 2.38440185282456
C -0.98773947807660 8.26698384188608 1.82428812494733
Cl -1.03639928772909 9.30664368214518 0.43584760657143
C 0.17051897730483 7.54224692977398 2.07960902966051
H 1.05464972459388 7.61341131267158 1.42515302758279
Cl 2.87128207876435 7.80519676972438 0.12211556681248
Cl 1.56705476673810 5.77720796897182 3.60160633194633

14m: $C_6H_6F_5$···Cl singlet
C 0.22589395623376 6.48038539293835 3.13034712323243
C -0.68627145741559 7.05576704648638 4.19845627564720
C -2.12796779882696 7.26403197101959 3.72338310742246
F -2.61320652689714 5.99272056376597 3.41279052316818
C -2.15204952875262 8.05389588628020 2.41070904492213
F -3.42995008567198 8.08993875444728 1.93482678852494
C -1.23855987322483 7.48244778483200 1.34151725060749
H -1.64100703718107 6.50818801354900 1.05294370430693
C 0.19028844930210 7.34044211372428 1.86674585566482
H 0.59936417351219 8.33036549025576 2.08652209821385
H 1.23348179476002 6.41847868769067 3.54861405884466
H -2.76309152334754 7.72879397664912 4.50221054285206
F -0.68810077879255 6.22575404724011 5.28094450043910
F -0.17771287666737 8.25242191664230 4.62318570308059
H -0.11999075327411 5.46621839740042 2.91526115316128
H 0.82379569131913 6.89187960589875 1.09658000912633
F -1.79000953396100 9.34874925813931 2.66238690589805
H -1.28541928369183 8.14441550298903 0.47333771286866
Cl -4.15090700742257 8.54192559005125 6.07865764201877

15m: $(CF_3)_3CH$···Cl singlet
C 0.12607144308261 -0.19243133157584 -0.07029874790174
C 1.65695248746701 -0.18199547632300 -0.12770663318460
H -0.23833498062844 -0.09501437426350 -1.13622282728691
C -0.43096732663280 1.01082391779452 0.69694453398619
C -0.42190102670465 -1.52026869914633 0.46230682104943
F -0.02030762621574 2.16736275156431 0.16446449555932
F -0.05364217984289 1.02768973031901 1.99841079787808
F -1.76910814777507 1.02484650708577 0.68617002824766
F 0.01868661059384 -2.55870790276712 -0.25778125751440
F -1.75889266221915 -1.55214372466178 0.42244890448056
F -0.06656912006034 -1.76122055639450 1.74768208778176
F 2.12246445228508 0.95828785035690 -0.65091414070351
F 2.12681775796151 -1.18264095234757 -0.88120142411044
F 2.23276496615363 -0.31160343662604 1.09216524710514
Cl -0.89275464746464 0.09065569698519 -3.06943788538653

16m: C5H4NC(O)H···Cl singlet
Cl 9.45726670759365 10.66899394524958 9.82160805196304
H 9.45973343810040 12.91999500667414 8.21019899555877
H 9.19873555835181 12.89273187739576 10.49368542298218
C 9.40865033691461 14.01997541423748 8.10089560415375
C 9.13805243165970 13.98251611685532 10.56199212040656
O 9.49000567130510 14.57241591383833 7.02935021307353
C 9.22986207225471 14.74990614140419 9.40480417639577
C 8.97311496277296 14.64600708846951 11.77187567240984
N 9.16868481700467 16.08680848196978 9.38804189234114
H 8.89807662516067 14.08222607429149 12.69625392708802
C 8.90805972420128 16.02943094163139 11.77066637735038
C 9.01117792492134 16.69900410813113 10.55229547306217
H 8.78023436477760 16.59223084759095 12.68980575921827
H 8.96351536498133 17.78636804226070 10.52198631399640

HL
O 4.00496067551386 6.16936427499176 4.74515322685584
N 6.33066664023567 6.17629982140105 3.26798344351098
N 8.63345951740948 4.62529860582930 3.63822675935839
C 4.11492607684851 6.88417513760370 3.77913415866039
N 5.25735498897119 6.95701131550782 3.00406218776201
C 7.37207834388426 6.39828911673716 2.57734514977370
C 8.61399731325310 5.61709066208840 2.74536537463518
C 9.75977953892184 3.93991445774042 3.77397940725955
C 3.01426393379951 7.79713788911373 3.31666798455582
H 5.33900577259828 7.69895548380834 2.32062738746671
H 7.39958826198846 7.18912648172296 1.81658063795178
C 9.71847524253639 5.94871566706703 1.96286871283125
C 10.91646373282794 4.19393459847399 3.04147260283145
H 9.74293358170112 3.13934788529702 4.50946718760774
C 2.13932105007373 8.28521124110372 4.28333184592016
C 2.82062680332999 8.13479490145046 1.97931599703231
H 9.65608091447263 6.76292606704045 1.24892483152055
C 10.89059049646988 5.22307366024199 2.11519968187426
H 11.80669403168635 3.59657699610104 3.19950948946266
H 2.29011699900111 7.99135984204622 5.31547003092876
C 1.09712569157354 9.12396473041500 3.91993387099897
C 1.76899748931124 8.96582729726903 1.61466327555974
H 3.46205959835422 7.72152361551266 1.20778639458372
H 11.76641314473101 5.45901101048440 1.52032053833172
H 0.42343516724173 9.50818039728761 4.67803564932510
C 0.91152058635449 9.46688838937274 2.58499953056084
H 1.61424697114487 9.21394878273549 0.57027917801052
H 0.09195483941077 10.11792628717113 2.30022062118878

$\{HL \cdot Cl\}^-$ assembly
O 4.16717784948320 6.48829388537722 4.87139312890547
N 6.39533979159467 6.29862839488957 3.34213485723674
N 8.76684104070169 4.86411604123151 3.87429915646012
C 4.22683269048661 7.07592535072541 3.80651700249046
N 5.31214173385566 7.03527101664064 2.97749509749426
C 7.35838642841367 6.31917533951851 2.50746063069813
C 8.60641431680946 5.57264383577473 2.75173857620677
C 9.91138800043270 4.21372801761404 4.03831733726403
C 3.06425787380086 7.91549439635905 3.32525526515027
H 5.35489871207291 7.53829328534061 2.07934726242766
H 7.29654818381919 6.88980112200028 1.57702413118680
C 9.59820689298480 5.64202743849286 1.76725731268397
C 10.95427822118530 4.22164149176235 3.11886228061958
H 10.00630089598745 3.64806296498192 4.96362683848708
C 1.96610060663167 7.99273720070853 4.18122106973192
C 3.03249010153474 8.59313218603655 2.10797799712590
Cl 5.93008259635433 8.37927538438478 0.10810484364089
H 9.41810038426151 6.23100940612388 0.87464592776726
C 10.78625193895989 4.95776500385990 1.95416995218989
H 11.86528114356792 3.66735450745711 3.31566226938672
H 2.01575368445196 7.45571020569763 5.12094656269955
C 0.84895467807147 8.73521991015388 3.83250468736878
C 1.90919521697667 9.33669814225533 1.76183314425564
H 3.86470284688330 8.56226921272056 1.40932548145295
H 11.56903520578876 4.99776557749613 1.20311938440867
H 0.00055196485125 8.78768872833524 4.50800672134283
C 0.81846916317392 9.41132431989427 2.61691356269391
H 1.89775593202236 9.85962200607123 0.81113165286757
H -0.05451809515831 9.99447562809580 2.33873786575598

CH_3NH_2 singlet
N -1.99504238810519 0.59816882436616 0.06043388973170
H -0.98299591509379 0.57750545985765 0.05288233045692
H -2.31389815603505 -0.13738374184621 -0.55768830593442
C -2.49339021165443 1.90063545936011 -0.37855047150572
H -2.14953480872535 2.67045084031045 0.31643749855862
H -3.58547094941747 1.89866417483238 -0.34410175059950
H -2.18558757096870 2.19541898311945 -1.39173319070761

$\{CH_3NH_2 \cdot Cl\}^-$ singlet
N -2.15831420528928 0.56394074202398 0.16104180383203
H -1.14200582466919 0.54894184534192 0.29803441830402
H -2.31838031838439 -0.12019627941140 -0.57114630631561
C -2.49729493572118 1.88792255193347 -0.35678999286563
H -2.33053206952541 2.63270314561353 0.42688388600053
H -3.55932854688728 1.92829059938840 -0.62986931685276
H -1.89576027483109 2.19172966273811 -1.22486779492477
Cl 1.12828617530784 0.94707773237196 -0.24583669717780

Dimer of 1 (triplet) optimized at the ωB97X-D4/ma-def2-TZVP (for Cu atoms)/ma-def2-SVP (all other atoms) level.
Cu 6.30674499693202 7.04215157734862 3.21817841177655
Cl 4.88176192010265 8.10485345361805 1.82718783578464
O 9.76716934641924 5.46169522660539 4.50079536282334
O 7.65708600064215 6.16453013898091 4.40264047002689
O 6.32785391118949 8.83416325330446 4.87010109451188
N 7.96957678609657 7.31521065266889 2.07795604505522
N 4.85262798735394 6.66162297805848 4.61660553973348
N 4.47198844860096 7.66169582324648 5.43775368476723
H 3.73632777257179 7.45056780318908 6.12302782926272
N 5.64835658218616 4.79170101677437 2.83643868597200
C 8.04843487298156 7.96414440065822 0.91536415344640
H 7.10697266001458 8.37882757798365 0.54331391983642
C 9.26345033644832 8.09434738523522 0.24102146271057
H 9.30046692764875 8.63521984477234 -0.70722217328494
C 10.41043345788195 7.52875459754879 0.79783246782556
H 11.37453933651529 7.61793245853213 0.28880511521012
C 10.31396015309133 6.84877038194364 2.01371418197904
H 11.17058523565539 6.38267004568809 2.50436053789024
C 9.06768827734003 6.77088913667409 2.62273049386958
C 8.84986860042306 6.07011792751473 3.95135817552748
C 6.11268245315451 3.85868641622742 2.01695097941150
H 6.83736651632440 4.18872990692225 1.26447833051456
C 5.71422780613851 2.52072039224069 2.09006582087461

H 6.12824665751753 1.78427566318717 1.39866669526085
C 4.79287641741054 2.15856566183332 3.06813048877350
H 4.46400337048706 1.12116710422712 3.16525148739991
C 4.30217162434881 3.13880426409757 3.93087471306983
H 3.59120968612683 2.89604795006791 4.72375133259196
C 4.75974141148847 4.44795439365393 3.77731662009396
C 4.28897138896053 5.52008694299910 4.67809422802176
H 3.48276154984111 5.31512857760622 5.38929809717897
C 5.32425811362811 8.72790150065355 5.56221804995150
C 4.96199638927363 9.76574366019821 6.58306785485105
C 5.63817724663619 10.98970357193367 6.50330291278852
H 6.37432160608754 11.12593990230154 5.70805105183912
C 5.36628865991899 12.00458757032240 7.41769532427738
H 5.88930615611585 12.96219721313925 7.34163673440180
C 4.42149181474633 11.79919801693554 8.42719609238776
H 4.20489959139347 12.59531696909771 9.14584328849112
C 3.75051623900722 10.57852161072481 8.51320642091724
H 3.00967406658002 10.41305117528798 9.30037873913799
C 4.01381165598814 9.56205392400310 7.59297094859288
H 3.46942479588059 8.61972514140307 7.68194123051503
Cu 6.15151370631249 4.91360108022127 8.77356645347657
Cl 7.57657608110372 3.85120643479131 10.16470985756014
O 2.69097721376189 6.49367878633959 7.49077651876511
O 4.80112412647223 5.79103341983716 7.58902065865293
O 6.13040488650035 3.12141778718978 7.12184951545622
N 4.48866969067552 4.64042912692102 9.91374855189659
N 7.60561255045488 5.29401691554032 7.37507097997111
N 7.98618901792062 4.29390075485956 6.55395724587514
H 8.72182599466406 4.50497449084410 5.86863944978595
N 6.80989091055351 7.16405262460401 9.15509734585424
C 4.40982893388369 3.99152228858505 11.07635605495699
H 5.35132548776045 3.57697845824749 11.44847531210003
C 3.19479163330466 3.86117710696873 11.75063215753853
H 3.15779161430253 3.32032290740130 12.69888695129846
C 2.04776587562947 4.42660245271323 11.19374061394300
H 1.08364261346756 4.33731277424348 11.70271546971804
C 2.14422178954695 5.10656654271745 9.97784591294410
H 1.28756523908080 5.57254723104258 9.48714038657731
C 3.39051604414035 5.18458752358854 9.36889406578987
C 3.62321308813940 5.88534877283355 8.04025541521277
C 6.34564491990676 8.09709737003701 9.97459444553162
H 5.62090886523702 7.76711570698936 10.72704386671686
C 6.74423597855633 9.43502553200937 9.90150905749689
H 6.33028067240472 10.17149830604958 10.59291679621269
C 7.66562430789736 9.79710563541817 8.92345048975794
H 7.99458736573677 10.83447803693424 8.82634598900447
C 8.15625218290714 8.81683227806359 8.06070350482574
H 8.86724385534650 9.05953530841178 7.26783810605689
C 7.69856936657067 7.50772115401524 8.21425163202339
C 8.16929367300953 6.43553872853756 7.31351238523184
H 8.97550305469976 6.64044052836465 6.60229049893096
C 7.13392994759451 3.22766268495272 6.42962921550045
C 7.49610892420573 2.18977657481347 5.40879654079029
C 6.81992617596783 0.96582359747816 5.48865204739259
H 6.08385126600648 0.82961159559223 6.28397215844678
C 7.09171314929522 -0.04907905476719 4.57425258766192
H 6.56868531952925 -1.00667844913500 4.65037401430519
C 8.03640086574384 0.15628475217949 3.56464296940797
H 8.25290474908263 -0.63984557025557 2.84598161122139
C 8.70739289924092 1.37694796052786 3.47855455273339
H 9.44816745057389 1.54239349280967 2.69131362601202
C 8.44422564308717 2.39342190545364 4.39882205545530
H 8.98862004257276 3.33573923661892 4.30978021584087

S66: model 01
O -0.69666003216128 -0.05047601702340 0.00979440023085
H -1.04628612193167 0.84384953376012 -0.01138271444632
H 0.26655326271274 0.04835831880480 0.00505371899032
O 2.23053436241059 0.02019783946644 0.00077488887738
H 2.59748871048786 -0.41258705464018 0.77782652158327
H 2.59300073548176 -0.45094826336778 -0.75581096023549

S66: model 03
O -0.66785317548817 -0.09909351202156 -0.01983961778279
H -1.05347799146011 0.77971453506992 0.01213093071382
H 0.29986804359625 0.03409488005862 -0.00324560696897
N 2.24722442757466 0.09453023549708 0.00595671040950
H 2.52025582214507 -0.46137050423588 -0.79610338638134
H 2.53489032354785 -0.41910127255488 0.83069753537305
C 2.88710674038744 1.41255157704191 -0.03444120371047
H 2.57030933187187 1.99288151311134 0.83484296035980
H 3.98347883016915 1.37969447417853 -0.04405743522734
H 2.55332753965599 1.94686505285492 -0.92654299178526

S66: model 04
O -0.38909615125857 -0.38376295352242 0.07183631581757
H -0.94416540536527 0.39250077186676 0.17673029850871
H 0.52883691347054 -0.05647334722332 0.09120879805458

C 2.19082995017605 -2.24724890217821 -0.22963158024740
H 2.84070668041691 -3.11338075757138 -0.36519444344596
H 1.50611754466303 -2.16592138548644 -1.07514107446293
H 1.57693456926788 -2.38500944798798 0.66173530149245
C 2.95286223805934 -0.94907684466959 -0.09678791459328
O 2.37759934375660 0.12260758949273 0.06077444835584
N 4.30212896706028 -1.03978460865714 -0.16176667893721
H 4.70882637631272 -1.94902491394758 -0.29239275708933
C 5.17919703532760 0.11038827621958 -0.05291018306147
H 4.55664439931106 0.99257435086701 0.08319389665011
H 5.84825365107271 0.01057294058598 0.80553013157763
H 5.77660201372911 0.23236551921200 -0.95997989161931

S66: model 09
N -0.87476660567196 0.00858453380001 -0.04222196185087
H 0.10165930814471 -0.25930856800099 -0.10018924313416
H -1.42620749326698 -0.83921550716166 -0.08524858797461
C -1.13337716729649 0.72549267752985 1.20327798201867
H -2.19141690982601 0.99454141844261 1.25960153560555
H -0.87752600391529 0.17009298722198 2.11803638568368
H -0.56447612824540 1.65907055509608 1.20865154021273
O 2.31727953099715 -0.09466987744824 0.05011970951812
H 2.68711235150092 0.17361632100378 -0.79514656720760
C 2.61147236261165 0.90785023893502 1.01500960138095
H 2.17161845541860 0.58015597566671 1.95746175217136
H 3.69123906826496 1.02881797695335 1.15727091086267
H 2.17307945628413 1.87485214796149 0.74318922071350

S66: model 18
O -0.53214269255377 -0.09666572716750 -0.00042778107746
H -0.87526085876893 0.80034750569795 0.00005625459439
H 0.43884939328330 -0.00281908498566 -0.00019855870004
N 2.37209667091899 0.09612318516801 0.00014623926481
C 3.05817983369185 0.06208877951257 1.14157636100528
H 2.46871409744604 0.08518735455043 2.05392003063075
C 4.44352362566861 -0.00232999293568 1.19395750647645
H 4.95165256517636 -0.02811375826061 2.15064618515903
C 5.14943778823545 -0.03486409681276 -0.00001845239485
H 6.23300499714479 -0.08604465405657 -0.00008195854490
C 4.44338169472924 -0.00236943350923 -1.19391088389862
H 4.95139537971402 -0.02818476084659 -2.15065987788207
C 3.05804383152384 0.06205130478324 -1.14136563546452
H 2.46846599179021 0.08512060386240 -2.05363782016825

CH_3OH
O 2.28738173338623 -0.04236537182213 0.02688053474141
H 2.56310978897506 0.31568527854386 -0.82064440303785
C 2.67039747441153 0.86416349448726 1.04957353120125
H 2.34456199980702 0.43080347016334 1.99580401549601
H 3.75749899264078 1.00371555762181 1.08806623414209
H 2.18888633477937 1.84247303300586 0.93208192745708

$O=C(CH_3)NHCH_3$
C 2.18999512364787 -2.24937466290466 -0.23040942274666
H 2.83833077452472 -3.11765724473024 -0.36371412869252
H 1.51392086602625 -2.17085708391615 -1.08326424511284
H 1.58070910347419 -2.38910753725519 0.66392020937082
C 2.94585235778029 -0.94255162686025 -0.09615097464826
O 2.36543374825141 0.11675202290199 0.05927379894778
N 4.30251346077306 -1.04080386926412 -0.16194833327291
H 4.70965193163308 -1.94957140907041 -0.29184785114801
C 5.17610693246020 0.11048187431192 -0.05285310954558
H 4.54822932557326 0.98948318317515 0.08243186610578
H 5.84547268720281 0.01640146139708 0.80649218156004
H 5.77463729865283 0.23752257021490 -0.95895678681765

Pyridine
N 2.36861238341346 0.09377816526171 0.00015231170715
C 3.06085905095812 0.06147453437410 1.13729364989170
H 2.47619108892262 0.08877873317136 2.05338395289829
C 4.44751728933791 -0.00319164764587 1.19258769179656
H 4.95493276433916 -0.02677609788910 2.15013319830133
C 5.15534482262954 -0.03622092910379 -0.00001684834422
H 6.23912010543730 -0.08660621637421 -0.00008247862147
C 4.44737060714223 -0.00323875723259 -1.19253546424579
H 4.95466742017603 -0.02686128759761 -2.15014286570144
C 3.06071894542948 0.06143037491571 -1.13707269480733
H 2.47593900021414 0.08869789312028 -2.05309264587479

Water
O -0.69620584320214 -0.05094626786596 0.00980209668705
H -1.03216150564934 0.84968885091878 -0.01153636426328
H 0.26149913285147 0.03409443894718 0.00540681457622
